# Financial motivation models for community health workers in low- and middle-income countries: a scoping review

**DOI:** 10.1080/16549716.2025.2480412

**Published:** 2025-04-04

**Authors:** Oumar Mallé Samb, Mariétou Niang, Emilie Gelinas, Ndeye Thiab Diouf, Titilayo Tatiana Agbadje, Abir El Haouly

**Affiliations:** a Health Research and Training Unit, Rouyn-Noranda Campus, Université du Québec en Abitibi-Témiscamingue, Abitibi-Témiscamingue, QC, Canada; b Department of Psychosociology and Social Work, Université du Québec à Rimouski, Lévis, QC, Canada; c Department of Social and Preventive Medicine, Laval University, Quebec, QC, Canada

**Keywords:** Community health workers, financial motivation, remuneration, impact, scoping review, low- and middle-income countries

## Abstract

Community health workers (CHWs) are key players in providing primary healthcare in low- and middle-income countries. However, their absence from the formal health system in many of these countries often presents a challenge to their remuneration. The objective of this scoping review is to document programs implemented at both small and large scales in low- and middle-income countries, the remuneration strategies they have established, and the effects of these strategies on the work of CHWs. In total, we included 50 articles in this review. We have identified four types of compensation: fixed compensation, performance-based compensation, compensation based on income-generating activities (IGAs), and combined compensation. We identified the strengths and weaknesses of each type of compensation. A common strength for most models was improvement in motivation and performance. A common weakness for most models was irregular payments. The results of this review highlight the need to consider the economic, social, and cultural settings of the countries or environments at hand, and to include CHWs in discussions regarding the selection of a compensation model.

## PAPER CONTEXT

- **Main findings**: Four financial motivation models for community health workers were identified in 24 low- and middle-income countries across three continents: fixed compensation, performance-based compensation, compensation based on income-generating activities (IGAs), and combined compensation.
- **Added knowledge**: This study reveals that there is no one-size-fits-all compensation model, and that the choice of model must be made based on the local economic, social, and cultural context, and through active collaboration and discussions with community health workers.
- **Global health impact for policy and action**: The findings of this review provide guidance to low- and middle-income countries seeking to introduce financial motivation models for community health workers, a strategy that could help improve the motivation, performance, and retention of these health workers.

## Background

Community Health Workers (CHWs) serve as liaisons between the community and the health and social services system [1,2]. Their titles and roles may vary across regions. Since the Alma-Ata Declaration in 1978, CHWs have been recognized as fundamental pillars of primary health care [3]. Many studies have demonstrated their value in reducing mortality and morbidity from certain diseases, improving accessibility of care, and strengthening ties between communities and health care services [1,4].

Despite their importance in the health system, CHWs face several challenges. In many low- and middle-income countries, CHWs are not part of the national health system and are regarded as volunteers. Furthermore, the work structure of CHWs has undergone profound transformations since the Alma-Ata conference. Such transformations include increased female CHWs, increased workloads [5], and adopting a model focused on the ongoing delivery of health services rather than one driven by community development [6]. Thus, volunteerism proves non-beneficial to CHWs as it caters instead to the requirements of health system constraints in terms of budgets and human resources [7,8]. In Ethiopia, the government opted for female CHWs, rather than men, not only because of their role in society but also to encourage volunteering as women are less likely to expect to be paid [7]. The sheer complexity of the global and local economic and sociopolitical contexts in which CHW programs are deployed makes it impossible to single out any one model of volunteering. Such complexity calls for a deeper reflection on workable, sustainable compensation models that align with the contextual realities of low- and middle-income countries.

Challenges associated with the ambiguity surrounding the place and status of CHWs in health service networks, bring forth the issue of compensation [9–12]. CHW incentives are critical in driving motivation, retention, and performance. There are several types of incentives, including financial incentives (e.g. salary) and non-financial incentives (e.g. goods) [12]. One study by Singh (2015) identified five different CHW compensation models in large-scale programs [13]: part-time volunteer CHWs without financial incentives, volunteer CHWs with financial incentives, full-time and part-time volunteer CHWs, volunteer CHWs selling health products, and full-time paid CHWs. Singh’s study laid the groundwork for further work aimed at understanding existing large-scale compensation models. However, this case study does not cover all large-scale programs, nor does it include small-scale programs. Also, the study’s incentive classification system does not specify the types of financial incentives and the underlying strategies.

The World Health Organization (WHO) recommends compensating practising CHWs [14]. For the purpose of this study, the term ‘compensation’ means a financial package based on the requirements and complexity of the work performed, the number of hours of work, training, and the roles played [15]. The WHO recommendation would enable the creation of more sustainable CHW programs within a country or geographic area. Yet, according to Ballard et al., many common CHW payment models do not reflect the WHO compensation recommendations [16]. Therefore, the objectives of this scoping review are as follows: 1) to describe the small- and large-scale CHW compensation strategies in low- and middle-income countries and their implementation methods, and 2) to describe the strengths and weaknesses of such strategies on the work of CHWs. Our research questions are as follows: What types of compensation are set up in the small- and large-scale CHW programs implemented in low- and middle-income countries? What strengths and weaknesses do these types of compensation have on the work of CHWs?

## Methods

To achieve the above-mentioned objectives, we conducted a scoping review. Our method is based on the methodological framework developed by Arksey and O’Malley [17]. This framework proposes the following five steps to conduct a scoping review: (1) identification of the research question; (2) identification of relevant studies; (3) selection of studies; (4) charting the data – data collection and analysis; and (5) collating, summarizing, and reporting the results [17].

This review uses the Preferred Reporting Items for Systematic Reviews and Meta-analyses (PRISMA) guidelines [18].

### Search strategy

We used several strategies to identify relevant articles and published documents [19]. We conducted a systematic literature search to identify published studies in the following electronic bibliographic databases: Embase (Pubmed), CINHAL and Google Scholar. We launched our search strategy in these databases in August 2023. We did not set a publication date limit. However, the first article identified was published in 1993. The search terms were adjusted in each database (see Supplementary File 1). Common search terms used were community health workers, community health aides, health volunteers, community health care, motivation, incentive, remuneration, salary, payment, and the names of the low- or middle-income countries. We performed a second round of searching within the references of relevant articles identified in these databases. We also conducted a search in the grey literature on the websites of international organizations (e.g. CHW Central, WHO) and low- and middle-income country governments. We performed these searches concurrently and incrementally, adjusting the keywords as we went to target countries or interventions in the grey literature search properly. These strategies were complemented by expert suggestions.

Author MN developed the search strategy with the help of an information specialist and carried out the search for articles and documents in the various databases.

### Study selection and eligibility criteria

Four authors (EG, MN, NTD and TTA) independently selected the articles using the Covidence platform [20]. The author OMS resolved any conflicts or discrepancies in terms of selection. We followed a two-phase process for selection: 1) reviewing titles and abstracts and 2) reviewing full texts. The predefined inclusion/exclusion criteria guided article selection.

We defined inclusion according to the PICOS model [21]: Population (P) – any descriptive or analytical study involving the work of CHWs; Intervention/Exposure (I) – existing small- and large-scale CHW compensation strategies; Comparator (C) – no restrictions; Outcome (O) – effects of compensation on the work of CHWs; and Study Design (S) – no restrictions. Low- and middle-income countries are the target of this review. We identified such countries using the World Bank country classification chart [22]. We only included articles published in French or English in this review.

We used the following exclusion criteria: all concept or theoretical papers unrelated to any existing compensation strategy or exploring CHW preferences in terms of compensation, and all papers that do not specifically address a well-defined CHW compensation strategy.

### Data charting

We extracted the following information into a spreadsheet: the general characteristics of the article (country, year of publication, type of publication, research design, study target population), the type of compensation strategy, the source of funding, the scope, the complementary strategies, the health care setting in which the intervention was developed, the local name given to CHWs, and the impact of compensation on the work of CHWs. MN, NTD, and EG followed a double-article extraction method.

### Data analysis

Using data processing software (Excel), we performed a descriptive analysis and narrative review for each category of extracted data.

## Results

### Article selection process

**Figure 1.**
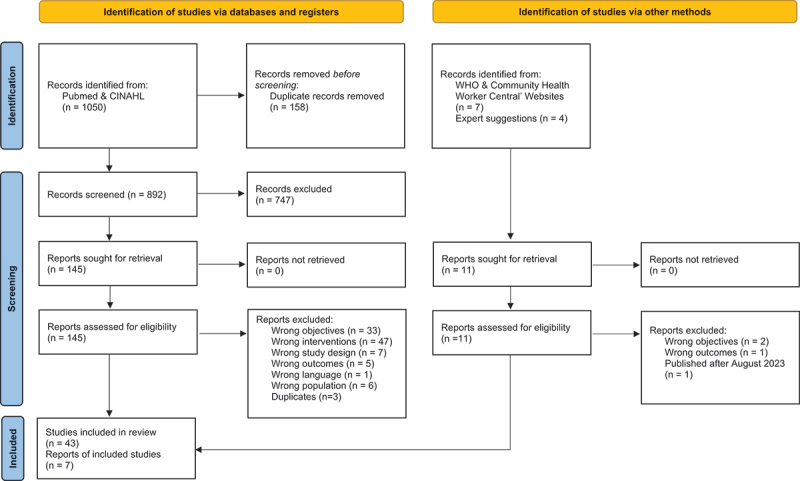
PRISMA flow diagram of the selection process.

Our search strategy enabled the identification of 1050 potentially eligible articles. After eliminating any duplicates (*n* = 158) and reviewing both the titles and abstracts of these articles (*n* = 892), we had a total of 145 eligible articles. Following the review of the full texts, we retained 43 articles. Searches conducted in other databases (including the WHO website and the websites of the government departments of various low- and middle-income countries) complemented by expert suggestions served to identify seven additional articles. We included 50 articles in this review [23–72]. Figure 1 shows the details of the selection process.

### Description of selected articles

Supplementary File 2 shows a description of the studies included in this review (first author name and the reference of the study, year of publication, country/countries, information on the CHW program, study design, type of study, CHW intervention area, type of remuneration, study participants). The 50 studies were conducted in 36 of the 78 countries classified by the World Bank as low- or middle-income countries [22]. Among these articles, 43 were peer-reviewed studies and seven covered grey literature [66–70]. Some articles focused on more than one CHW program [52,54,69,71,72].

Table 1 presents the countries in which the studies were carried out or to which the document refers, the number of programs related to each country, and the areas of intervention of the CHW program that we grouped into four categories. We included hygiene interventions in the health promotion and disease prevention category.

**Table 1.** Description of intervention areas by country.

		Areas of intervention				
Countries		n	Maternal, neonatal and child health (including ICCM)	General primary health care	Sexual and reproductive health services	Health promotion prevention and disease surveillance
Africa						
	Afghanistan	1		[71]		[71]
	Benin	1	[72]	–	–	–
	Burkina Faso	2	[68,72]	[68,72]	[68,72]	[68,72]
	Cameroon	1	–	–	–	[39]
	Ethiopia	4	-	[25,36,54]	[71]	[71]
	Ghana	5	[51,58,67,71,72]	[51,67,72]	[71,72]	[67,72]
	Kenya	4	[52,71]	[42,43]	–	–
	Liberia	2	–	[66,71]	–	–
	Madagascar	2	[69]	[69]	[69]	[69,71]
	Malawi	4	[52,71,72]	[71,72]	[71]	[54,71,72]
	Mali	1	[72]	[72]	[72]	[72]
	Mozambique	3	[32,54]	–	–	[32,54,71]
	Myanmar	1	–	[71]	–	[71]
	Niger	2	[72]	[71,72]	[72]	[72]
	Nigeria	4	[49,52]	[53,71]	–	–
	Uganda	1	–	[50]	–	–
	Rwanda	2	[71]	[33,71]	–	–
	Senegal	2	[70,73]	–	–	–
	Sierra Leone	1	[71]	[71]	–	[71]
	South Africa	1	–	[71]	–	[71]
	Tanzania	5	[41,62]	[71]	[46,47]	[62,71]
	Zambia	3	–	[65,71,72]	–	[65,71]
	Zimbabwe	2	[30]	[71]	–	[71]
Asia						
	Bangladesh	8	[23,24,52,71]	[56,57,63]	[37,57,71]	[56,57]
	India	10	[26,27,45,52,59–61,71]	[48,55,71]	[71]	[61,71]
	Indonesia	2	–	[54]	–	[38]
	Iran	1	[71]	[71]	[71]	[71]
	Pakistan	2	[71]	[44,71]	–	[71]
	Papua New Guinea	2	–	[29,31]	–	–
	Nepal	1	[71]	[71]	–	[71]
	Philippines	2	–	[35]	[34]	–
	Thailand	1	–	[71]	–	[71]
	Vietnam	1	–	[74]	–	–
America						
	Brazil	1	–	[71]	–	–
	Guatemala	1	–	–	–	[71]
	Haiti	1	–	[40]	–	–

ICCM = Integrated community case management.

### Mapping the compensation strategies and their implementation methods

We identified four types of compensation: fixed compensation, performance-based compensation, compensation based on income-generating activities (IGAs) and combined compensation.

#### Fixed compensation

In the fixed compensation model, CHWs receive a set amount every month. This model proposes two payment systems: salary-based and monthly motivation. The difference resides in the degree of formalization of the CHW in the hiring structure. The salary-based compensation comes with obligations concerning the CHW mandate (number of hours, days, etc.), which is not the case for the monthly motivation compensation where there are fewer expectations on CHWs’ work.

Forty CHW programs related to 17 studies/reports use the salary-based compensation model [24,25,36,40,41,45,48,49,52,54,56,57,64,65,67,71,72]. CHWs are employed by the government [25,36,45,48,49,54,57,65,67,71,72,75], national and international organizations and/or initiatives (including research projects) [24,40,54,56,64,71,72], and both the state and a non-governmental organization (NGO) [41,71,72]. In studies that reported the information, CHWs are part-time [56,71] or full-time contract personnel [24,40,64,67,71,72]. In all the programs featuring salary-based compensation, the CHWs received a set amount through a monthly salary that varied between US$20 [56] and US$380 [65] (See supplemental file 2). Nine studies reported the applicable recruitment criteria (e.g. speak the local language, must have no criminal or behavioural history, must be a woman, must be nominated by a distinguished member of the community such as a local leader) [41,48,49,52,56,64,65,67,71]. All the studies/reports reported the complementary measures offered to CHWs (e.g. supervision, continuing education, provision of equipment, preferential treatment) and specify the mandate of CHWs, who perform various tasks (e.g. promotion, prevention and curative activities).

Thirty-one CHW programs related to 13 studies/reports use monthly motivation-based compensation models [29–32,34,43,46,54,62,64,68,71,72]. In the motivation-based compensation, we included financial incentives (ex. allowances) and all types of remuneration that were not a salary. In all these programs, the CHWs were regarded as volunteers. For twenty-two programs, the CHWs were part-time volunteers [31,32,34,46,62,68,71,72]. In fifteen programs, the CHWs were employed by the government [31,32,34,54,68], sometimes in collaboration with other partners [71,72,76]. In the sixteen remaining programs, they worked with national and international organizations and/or initiatives [37,43,46,62,64,71,72], private companies and non-profit foundations [29], or research projects [30]. Their monthly motivation varied between US$5 [54] and US$70 [32] (See supplemental file 2). Nine studies reported the recruitment criteria for CHWs [29–31,34,43,46,64,71,72] (e.g. be 18 years of age or older, be well known in the community, have a basic level of literacy, have lived in the district for more than five years). All studies reported the complementary measures available to CHWs (e.g. refresher training, replenishment of supplies, mentoring, supervision) and specified the mandate of CHWs who performed various tasks (e.g. family planning services). The initial motivation-based compensation model of two programs changed after program implementation to a combined model due to changes in national policy for the first [29] and compensation based on IGAs for the second due to changes in governance leading to the decentralization of resources [43]. Two programs used salary-based and monthly motivation-based compensation models [64,71].

#### Performance-based compensation

Twenty-six CHW programs included in 22 studies/reports targeted in this review followed a performance-based compensation model [26–28,33,35,44,45,48,50–52,55,58–61,69,71,72,77–79]. This type of compensation includes service-based, activity-based, and performance-based incentives. In these types of compensation models, each program establishes a number of activities to perform, services to provide, or goals to achieve by the CHWs to be entitled to compensation. The CHWs are, therefore, paid according to their results measured using performance indicators. The CHWs in these programs are regarded as contract volunteers. These programs were initiated by government [26–28,45,48,52,55,59–61,71], national and international NGOs, and initiatives [33,35,44,51,58,69,71,72], and multiple governmental and non-governmental organizations [50,77–79]. Thirteen studies reported recruitment criteria for CHWs (e.g. social acceptance, recruited on a voluntary basis) [27,28,33,35,45,52,59,60,71,77–80]. Twelve studies reported that the amount CHWs receive depended on the act performed [26,27,45,50,55,59–61,69,71,77,78] (See Supplemental file 2). For instance, in the ASHA program, CHWs earn between US$2.14 and US$3 per immunization session [26,45], US$10 for facilitating an institutional delivery [45], US$0.83 for early registration of pregnancy, and US$16.67 for facilitating permanent contraceptive methods [60]. However, some sub-tasks among their responsibilities are unpaid [27]. In Indonesia’s SMART*health* program, *kaders* (CHWs) receive either a full monthly financial incentive after performing 100% of follow-up with their assigned patients, or US$1.1 per patient if all follow-ups are not completed. In the latter case, three unsuccessful attempts to reach and follow up on a patient is considered a successful follow-up [77]. One program set a maximum monthly compensation that CHW can earn [78]. Twelve programs reported that the performance-based incentive was paid monthly [27,44,45,50,55,58,60,71,72,77,78], and one quarterly [71]. One program set a maximum monthly compensation that CHW can earn [78]. All programs reported supervision as an additional measure offered to CHWs. Other measures included resources that supported job performance, such as drug-kit delivery, medical supplies, and the use of medical kits, bicycles, mobile SIM cards, and uniforms [28,44,55,59,69].

#### Compensation based on IGAs

Six CHW programs included in five studies/reports used a compensation model based on IGAs [43,53,69,70]. These programs are initiated by national and international NGOs and/or initiatives [43,69,70] and by the government [53,71]. In five programs, the CHWs were regarded as community volunteers [43,69–71]. IGA-based or loan system compensation are models in which (1) some or all CHWs have access to loans that can help them enter into an income-generating activity [43,69–71]; or (2) CHWs receive pharmaceutical donations, which they then sell and keep the profit [53]; or (3) CHWs receive user fees from the sale of drugs and products (compensation derivate from IGAs) [69]. Three studies reported the recruitment criteria for CHWs (e.g. must not hold a full-time paid job) [43,53]. The IGA can be linked to the project (e.g. selling antimalarial drugs) [53], or can be carried out independently (e.g. loan systems) [43,70,71]. Thus, the CHWs can perform activities unrelated to the project (e.g. animal husbandry) [70]. The activities carried out by the CHWs were specified in the studies (e.g. caring for patients with moderate cases of malaria). The estimate of the average monthly earnings of CHWs was only reported for one program (US$160) [53]. Five studies reported additional measures available to CHWs [43,53,69–71].

#### Combined compensation

Table 2 presents the different types of combined compensation. Twelve CHW programs use the combined compensation model [23,29,37,42,54,66,71,72,81]. This model combines two or more of the above-mentioned types of compensation. For example, in the depot-holders program in Bangladesh, the fixed compensation (monthly honorarium) is combined with compensation based on IGAs (50% of profits from the sale of commodities) and performance-based compensation (50% of service charge for customers they referred to the NGO clinics) [37] (See supplemental file 2). For one program using IGAs and motivation-based compensation, the incentives have been removed two years after the implementation of the program [81]. These programs are funded by national or international NGOs [23,29,37,54,81] or multiple governmental and non-governmental organizations [42,66,71,72]. Seven studies reported that the CHWs were volunteers [23,29,42,54,71,72,81]. Four studies reported the recruitment criteria for CHWs (e.g. must be a respected permanent resident of their village) [23,29,54,71]. Eight studies specify the additional measures available to CHWs (e.g. weekly follow-up meetings for CHW capacity development) [23,37,54,63,66,81].

**Table 2.** Types of combined compensation.

Authors	Fixed compensation		Performance-based compensation	Compensation based on IGAs
	Salary-based compensation	Monthly motivation-based compensation		
Alam et al. [24]		✔		✔
Burkot et al. [29]		✔ ^(removed in 2009)^	✔	✔
Devlin et al. [66]		✔	✔	
Gazi et al. [37]	✔		✔	✔
Kawakatsu et al. [42]	✔		✔	
Ormel et al. [54]			✔	✔
Tariqujjaman et al. [63]		✔		✔
PMI Impact Malaria [72]		✔		✔
Perry et al. [87]			✔	✔
		✔	✔	
	✔		✔	
	✔			✔

## Strengths and weaknesses of each type of compensation

In this section, we present the positive and negative effects of each compensation strategy identified in this review on the work of CHWs. Supplemental File 3 presents the strengths and weaknesses of each type of compensation.

### Fixed compensation

The positive effects of fixed compensation (salary and monthly motivation) are as follows: improved performance [74] and motivation [49,54,82], added prestige and value of CHWs within the communities [49], and career development opportunities. These factors contribute to CHW retention [30–32,65]. Also noteworthy is a heightened sense of belonging [43] and improved living conditions due to having regular and adequate wages [23,40,49,82]. Such wages are a leading indicator of the performance, motivation, and quality of services rendered by CHWs [24,45,48], allowing them to cover their work-related expenses [43].

Some factors which can have detrimental effects on CHW motivation and retention are: short-term contracts [32]; low wages [45,71]; irregular payments, especially in the case of NGOs [31,36,43,52,54,65,71]; perception of earning inadequate salaries and/or motivation/incentives (salary below US$25 in the targeted programs) for the workload and the environment’s socioeconomic realities [25,30–32,34,40,43,57,62,71]; confusion as to the limits of the tasks and roles assigned to CHWs, and confusion as to the possibility of having other jobs at the same time [43]; change in perception regarding their status (community agents versus government employees) [71]; preference for holding an employee status over a volunteer status due to stability issues [30,43]; and frustration stemming from the disparity between CHWs receiving salary-based payments and those relying on monthly stipends [54] or salary discrimination between CHW cadres [57]; and differential treatment based on the source of compensation [54]. Indeed, while CHWs who work in government facilities are entitled to *per diem* allowances, the same is not true for CHWs who work in NGO structures [54]. In the Bangladesh WASH program, CHWs received little support from their families and neighbours, particularly at the beginning of the project, because they felt they were poorly paid [82].

### Performance-based compensation

Performance-based compensation and additional measures such as training and supervision constitute a genuine source of motivation for CHWs [33,38,52,55,58,59]. The income earned by CHWs and the various financial motivations they receive kindle their interest in working as CHWs [44,55], grant them further autonomy regarding household purchases and management [33,38,58,60], contribute to their performance [38,58], and strengthen their ties with community members and women of childbearing age [59,61]. Furthermore, individuals working as CHWs can better contribute to health decisions [60] and assert greater authority within their households [33]. Performance-based compensation can be cost-effective, even with incentives for CHWs [26], favours low attrition rates, and is less susceptible to fraud, as CHWs are paid based on work completed [50].

This compensation system is competitive in nature [50,60,71] and can create inequalities as CHWs serving larger populations or easy-access areas earn more than other CHWs [55,59]. The factors that threaten CHW motivation and retention in performance-based compensation models are as follows: imbalance between the number of tasks at hand and the number of paid activities [26,28,50,52,55], result-based compensation rather than effort-based compensation [39,52,55], irregular payments [27,35,47,59,60] and low wages leading to quitting [27,28,50], negative perception of low wages by family members [27,28,50], complexity and delays in the payment request process [27,55,59,60], lack of time for family and other IGAs [59], lack of understanding of the compensation system, and downgrading of volunteer work by community members and family due to several indirect charges incurred by CHWs [27,59]. The excessive workload of CHWs is an obstacle to the sustainability of their activities [33]. Factors with adverse effects on the performance of CHWs are as follows: turning down certain non-paid activities (e.g. community engagement) to perform paid activities [59–61,71], perception among doctors and nurses that the selection of CHWs was influenced by favouritism and local leaders, which constitutes a source of frustration [59], difficulties faced by certain CHWs in correctly filling payment worksheets (which impacts compensation) [60], and insufficient remuneration to cover work-related expenses (e.g. transportation, communication, uniforms) [39,60].

### Compensation based on IGAs

The only positive effect reported for this type of compensation was satisfaction with the salaries received by the CHWs [43]. The negative effects, however, were linked to motivation, retention, and sustainability, including heavy workloads not adequately reflected in earned income [43]; large income gaps among CHWs, leading to frustration and loss of motivation [53]; significant disparities in the success of individual cooperatives in generating sufficient income for CHWs and broader income-generating activities [71]; an imbalanced distribution of time between activities, with more time allotted to IGAs than to volunteer work [43,53]; expenses and debt resulting from CHWs’ financial contributions to access the loan system [43]; and a lack of financial support from the State and local communities [70].

### Combined compensation

The factors at play in enhancing CHW performance and increasing their motivation to deliver quality services were compensation [29,42,63], varied sources of revenue allowing CHWs to compensate for certain work-related expenses and provide for their families [69], additional revenue from the sale of health products [29,63], supervision, and career development opportunities [69]. Other positive effects of this type of compensation were CHW appreciation of monthly motivations [42,54] and higher retention rates among the CHWs earning the highest average monthly incomes [23,42].

The negative effects of combined compensation are the perception among CHWs that they work hard while the program initiators receive all the credit on the national and international stage [29]; the perception that the health products sold are expensive [29], which leads to decreased community support [29]; lack of career development opportunities [69]; loss of motivation among CHWs due to different incomes for CHWs in the same program, irregular motivation payments among CHWs working for several agencies, and inconsistent supervision practices [69]; the prioritization of higher paid activities [63]; perception of insufficient salary support [71]; and the perception of having low wages as compared to the living standard in the community [37].

## Discussion

In this scoping review, we described the small- and large-scale CHW programs implemented in low- and middle-income countries, the compensation strategies in place, and the strengths and weaknesses of such strategies on the work of CHWs. These results lead us to make the following observations.

First, we identified four types of compensation: fixed compensation, performance-based compensation, compensation based on IGAs and combined compensation. While the first three types of compensation have been identified in other reviews [13,75,83], none of the studies mentioned combined compensation. Unlike these prior reviews, our study did not focus on non-paid CHW programs, as in two reviews (unpaid volunteers and part-time volunteer CHWs without financial incentives) [13,75].

The definition of compensation varies from one author to another. For instance, the nature of IGAs is diverse and could potentially be subcategorized. The same applies to salaries, as the conditions and mandates assigned to CHWs vary from program to program. This discrepancy suggests that the compensation models proposed here are not rigid frameworks, as there is no universal definition. Moreover, the selection of compensation types is not always justified in the studies targeted by our review to allow a global contextual analysis. The WHO guidelines on health policy and system support to optimize CHW programs [14] do not provide guidance for choosing the type of compensation. Thus, we recommend that program developers use a context-based participatory approach focused on CHWs and their needs by actively involving them in discussions to better identify the compensation model that would promote their engagement and fully empower them within their community.

Second, there are strengths and weaknesses for each model of compensation [71]. A common strength for most models is improvement in motivation and performance. A common weakness for most models is irregular payments. The latter has also been reported among the commonly shared incentives-related challenges in the compendium of 29 national CHW programs [71]. While the WHO favours an income-based model [14], payment irregularities raise questions about the sustainability of the wages and the programs, which depend on the financial capacity of the funding bodies. Unarguably, CHW program sustainability is influenced by strategic areas, such as CHW payment and program financing [32]. As shown by the relaunch of the official CHW program in Mozambique, dependence on external funding – especially when both external and government funding are declining – may hamper sustainability [32]. For example, in Senegal, in 2023, the government committed to pay the 9138 *Bajenu Gox* (which means ‘godmother of the neighbourhood’ in Wolof; community health volunteers) US$80 per month from the national budget, supplemented by a private foundation [84]. Two years later, the State’s contribution was still inadequate. This inefficiently jeopardizes the sustainability of the motivation mechanism, especially since the foundation only committed for one year.

Although it fosters competition among CHWs, motivating them to perform better [71], performance-based compensation can also be a source of inequality among CHWs as remuneration is based on the performance of each individual [55,59], and is result-based compensation rather than effort-based compensation [39,52,55]. Performance-based incentives do not provide financial security and ultimately impede CHW’s rights to better service conditions [28]. Additionally, the WHO suggests not paying CHWs exclusively or predominantly according to performance-based incentives [14].

Compensation based on IGAs could be a financial burden among CHWs as contributions to access the loan system are not always compatible with their standard of living and income [70].

CHWs’ profiles and assigned functions vary significantly from one program/intervention to the next. This variety influences the proposed compensation, making it difficult to recommend a single model for all low- and middle-income countries. Consequently, the results of this review suggest that one must consider the socioeconomic and cultural context of the target country/environment to select the type of compensation. This implies that each country’s definition of the role of CHWs should be taken into account, depending on whether this role is equated with self-giving, development, and community involvement. For instance, it might be difficult for some economically weaker countries to offer compensation in the form of salary, and especially ensuring its sustainability, is not possible without institutionalizing these programs within the country’s administrative routines (strong political commitment and sustainable financing) [85]. Moreover, seeing as some of the programs targeted herein have experienced a shift toward combined compensation [29,43], one can argue that compensation models are dynamic and can evolve over time. Regardless, combined compensation stands as an interesting option to explore.

Third, this review highlights the limitations of volunteerism and draws attention to the importance of compensation for CHWs. However, while compensation provides financial stability to CHWs, in certain circumstances, such as in programs with performance-based compensation, it may push some CHWs to turn down non-paid activities to pursue only paid activities. The literature clearly shows that the financial support given to CHWs produces unexpected consequences such as heightened interest in CHW positions offering allowances [80].

The idea of volunteer CHWs has evolved among the different programs studied, and there is a need to revisit the situation of CHWs in light of these learnings. This would involve formalizing the status of CHWs by restructuring basic notions such as their rights, profile and mandate. One example is the government of Ghana, which defined its CHW Program Conceptual Framework within the Ghana Health Service/Ministry of Health service delivery framework to strengthen healthcare delivery at the community level [67]. To ensure program sustainability, governments should thus define a clear national policy and the place of CHWs within the health system.

Fourth, the analysis of the impacts of the various types of compensation shows that compensation alone does not suffice to promote CHW performance, motivation, and retention. These three aspects depend on support measures such as training and supervision, which are reported to positively impact CHW performance and motivation. There is data to support that CHW training and skills development constitute one of the four essential levels of support and intervention identified in advancing the field of CHWs [86]. Furthermore, other authors found that supervision is critical for the effectiveness of CHWs and appears to be effective in combination with other supports [83]. In this same line of thought, Colvin et al. recommended multidimensional incentives to sustain CHWs motivation [87]. Therefore, to ensure the proper functioning of the programs, it is important that CHWs take advantage of beneficial approaches to training (e.g. mixing of training components) and supervision (e.g. focusing on supportive approaches) [83], and that program supervisors build a collaborative culture with CHWs rather than one based on subordination [80].

Fifth, in several programs, those dedicated to improving maternal, neonatal and child health, and those relating to sexual and reproductive health, common selection criteria included being a woman or a married woman with a certain level of education. While the selection may be based on social acceptability issues targeting increased social inclusion of women, one can also question the possible adverse effects of the feminization of the work of CHWs. This suggests that in the case of unpaid female volunteer CHWs, who already have a very full plate with their housework, this mandate and its related workload leave no room for other economic activities, thus reinforcing gender inequalities and contributing to disproportionately high poverty rates among women throughout their lives [88].

### Limitations

This study has several limitations. First, we included both small- and large-scale programs in low- and middle-income countries. While the review encompassed CHW programs implemented in 24 low- and middle-income countries across three continents, the conclusions of this study may be difficult to generalize due to the unique context of each country studied. Therefore, we suggest that the social and cultural contexts of the target country or environment be carefully considered before selecting any type of compensation. Second, we may have inadvertently overlooked one or more articles covering small- or large-scale CHW programs in low- and middle-income countries. However, we adopted a diversified search strategy to identify all published articles and grey literature on the subject. While this approach allowed us to identify numerous studies, it also complicated the comparison of results regarding the effectiveness of each type of compensation due to the diverse nature of CHW programs.

## Conclusion

In this scoping review, we documented the small- and large-scale CHW programs in low- and middle-income countries, the compensation strategies implemented, and the impact of such strategies on the work of CHWs. Our findings show that no single compensation model would fit every low- and middle-income country. The selection of a compensation model must be made based on the local economic, social and cultural context, and through active collaboration/discussions with CHWs. In addition, the compensation model can be dynamic and evolve over time. Moreover, compensation must be accompanied by support measures such as training and supervision built on proven beneficial approaches. The status of CHWs should be formalized and restructured by defining basic notions such as their rights, profile, and mandate. Each government should establish a clear national policy recognizing the place of CHWs within the health system. In conclusion, this review’s findings will guide low- and middle-income countries seeking to introduce or strengthen a CHW program.

## Supplementary Material

Supplementary file search strategy.docx

Supplementary file 3_Strengths and weaknesses_.docx

Supplementary file 2_Description of studies_.docx

## Data Availability

The datasets used and/or analyzed during the study are available from the corresponding author upon reasonable request.
