# Supplementary material for: Financial motivation models for community health workers in low- and middle-income countries: a scoping review
Source: Glob Health Action. 2025 Apr 4;18(1):2480412. doi: 10.1080/16549716.2025.2480412 (PMC11980195; doi:10.1080/16549716.2025.2480412)
Supplement: Supplementary file search strategy.docx [file ZGHA_A_2480412_SM3206.docx]

**Supplementary file 1**

| **Database** | **Search Strategy** |
| --- | --- |
| **PubMed** | community health workers[MeSH] OR community health aide*[TIAB] OR community health worker*[TIAB] OR health volunteers[TIAB] OR community health care[TIAB] "Motivation"[Mesh:NoExp] OR "Motivation"[TIAB] OR incentive*[TIAB] OR "Remuneration"[Mesh] OR "Remuneration"[TIAB] OR Salarie*[TIAB] OR payment[TIAB] egypt[MeSH:noexp] or morocco[MeSH:noexp] or tunisia[MeSH:noexp] or cameroon[MeSH:noexp] or central african republic[MeSH:noexp] or chad[MeSH:noexp] or congo[MeSH:noexp] or "democratic republic of the congo"[MeSH:noexp] or equatorial guinea[MeSH:noexp] or gabon[MeSH:noexp] or "sao tome and principe"[MeSH:noexp] or burundi[MeSH:noexp] or djibouti[MeSH:noexp] or eritrea[MeSH:noexp] or ethiopia[MeSH:noexp] or kenya[MeSH:noexp] or rwanda[MeSH:noexp] or somalia[MeSH:noexp] or south sudan[MeSH:noexp] or sudan[MeSH:noexp] or tanzania[MeSH:noexp] or uganda[MeSH:noexp] or angola[MeSH:noexp] or lesotho[MeSH:noexp] or malawi[MeSH:noexp] or mozambique[MeSH:noexp] or swaziland[MeSH:noexp] or zambia[MeSH:noexp] or zimbabwe[MeSH:noexp] or benin[MeSH:noexp] or burkina faso[MeSH:noexp] or cabo verde[MeSH:noexp] or cote d'ivoire[MeSH:noexp] or gambia[MeSH:noexp] or ghana[MeSH:noexp] or guinea[MeSH:noexp] or guinea-bissau[MeSH:noexp] or liberia[MeSH:noexp] or mali[MeSH:noexp] or mauritania[MeSH:noexp] or niger[MeSH:noexp] or nigeria[MeSH:noexp] or senegal[MeSH:noexp] or sierra leone[MeSH:noexp] or togo[MeSH:noexp] or honduras[MeSH:noexp] or nicaragua[MeSH:noexp] or bolivia[MeSH:noexp] or kazakhstan[MeSH:noexp] or kyrgyzstan[MeSH:noexp] or tajikistan[MeSH:noexp] or uzbekistan[MeSH:noexp] or cambodia[MeSH:noexp] or laos[MeSH:noexp] or myanmar[MeSH:noexp] or philippines[MeSH:noexp] or timor-leste[MeSH:noexp] or vietnam[MeSH:noexp] or bangladesh[MeSH:noexp] or bhutan[MeSH:noexp] or india[MeSH:noexp] or afghanistan[MeSH:noexp] or syria[MeSH:noexp] or yemen[MeSH:noexp] or nepal[MeSH:noexp] or pakistan[MeSH:noexp] or sri lanka[MeSH:noexp] or "democratic people's republic of korea"[MeSH:noexp] or mongolia[MeSH:noexp] or borneo[MeSH:noexp] or melanesia[MeSH:noexp] or papua new guinea[MeSH:noexp] or vanuatu[MeSH:noexp] or haiti[MeSH:noexp] or comoros[MeSH:noexp] or madagascar[MeSH:noexp] or sri lanka[MeSH:noexp] OR (Afghanistan[TIAB] OR Afghani[TIAB] or Afghan[TIAB] OR Angola*[TIAB] OR Bangladesh*[TIAB] OR Benin[TIAB] or Beninese[TIAB] OR Bhutan[TIAB] OR Bolivia*[TIAB] OR Burkina Faso[TIAB] OR Burkinabe[TIAB] or Burundi*[TIAB] OR Cabo Verde[TIAB] OR Cape Verde[TIAB] OR Cambodia*[TIAB] OR Cameroon*[TIAB] OR Central African Republic[TIAB] OR Chad[TIAB] or Chadian[TIAB] or Tchad[TIAB] OR Comoros[TIAB] OR Comoran[TIAB] OR Congo[TIAB] or Congolese[TIAB] OR Cote d'ivoire[TIAB] OR Ivorian[TIAB] OR Djibouti[TIAB] OR Egypt[TIAB] or Egyptian[TIAB] OR El Salvador[TIAB] OR Salvadoran[TIAB] OR Eritrea*[TIAB] OR Ethiopia*[TIAB] OR Gambia[TIAB] OR Gambia[TIAB] OR Ghana*[TIAB] OR Guinea[TIAB] OR Guinea Bissau*[TIAB] OR Haiti[TIAB] Or Haitian[TIAB] OR Hondura*[TIAB] OR India[TIAB] OR Indonesia*[TIAB] OR Kenya*[TIAB] OR Kiribati[TIAB] OR North Korea*[TIAB] OR DPRK[TIAB] OR Kosovo[TIAB] OR Kosovar[TIAB] or Kosovan[TIAB] OR Kyrgyz*[TIAB] OR Laos[TIAB] OR Laotian[TIAB] OR Lesotho[TIAB] OR Mosotho[TIAB] or Basotho[TIAB] OR Liberia*[TIAB] OR Madagascar[TIAB] OR Malagasy OR Malawi* OR Mali OR Malian OR Mauritania* OR Micronesia*[TIAB] OR Moldova*[TIAB] OR Mongolia*[TIAB] OR Morocco[TIAB] OR Moroccan[TIAB] OR Mozambique[TIAB] Or Mozambican[TIAB] OR Myanmar[TIAB] OR Burmese[TIAB] or Myanmarese[TIAB] OR Nepal[TIAB] OR Nepalese[TIAB] OR Nicaragua*[TIAB] OR Niger[TIAB] OR Nigerien[TIAB] OR Nigeria[TIAB] OR Pakistan*[TIAB] OR Papua New Guinea*[TIAB] OR Philippines[TIAB] OR Filipino*[TIAB] OR Rwanda*[TIAB] OR "Sao Tome and Principe"[TIAB] OR San Tomean[TIAB] OR Senegal*[TIAB] OR Sierra Leone*[TIAB] OR Solomon Island*[TIAB] OR Somalia*[TIAB] OR Sri Lanka*[TIAB] OR Sudan[TIAB] or Sudanese[TIAB] OR Swaziland[TIAB] OR Swazi[TIAB] OR Syria[TIAB] or Syrian[TIAB] OR Tajikistan[TIAB] OR Tajik[TIAB] or Tadzhik[TIAB] OR Tanzania*[TIAB] OR Timor Leste[TIAB] OR Timorese[TIAB] OR Togo[TIAB] OR Togolese[TIAB] OR Tunisia*[TIAB] OR Uganda*[TIAB] OR Ukraine[TIAB] OR Ukrainian[TIAB] OR Uzbekistan*[TIAB] or Uzbeki[TIAB] OR Vanuatu[TIAB] OR Vietnam*[TIAB] OR West Bank[TIAB] OR Gaza[TIAB] OR Yemen*[TIAB] OR Zambia*[TIAB] OR Zimbabwe*[TIAB] |
| **CINAHL** | MH "Community Health Workers" OR TI (community health aide* OR community health worker* OR health volunteers OR community health care) OR AB (community health aide* OR community health worker* OR health volunteers OR community health care) MH (Motivation OR “Salaries and Fringe Benefits”) TI (incentive* OR Remuneration OR Salarie* OR payment) OR AB (incentive* OR Remuneration OR Salarie* OR payment) TX (egypt or morocco or tunisia or cameroon or central african republic or chad or congo or "democratic republic of the congo" or equatorial guinea or gabon or "sao tome and principe" or burundi or djibouti or eritrea or ethiopia or kenya or rwanda or somalia or south sudan or sudan or tanzania or uganda or angola or lesotho or malawi or mozambique or swaziland or zambia or zimbabwe or benin or burkina faso or cabo verde or cote d'ivoire or gambia or ghana or guinea or guinea-bissau or liberia or mali or mauritania or niger or nigeria or senegal or sierra leone or togo or honduras or nicaragua or bolivia or kazakhstan or kyrgyzstan or tajikistan or uzbekistan or cambodia or laos or myanmar or philippines or timor-leste or vietnam or bangladesh or bhutan or india or afghanistan or syria or yemen or nepal or pakistan or sri lanka or "democratic people's republic of korea" or mongolia or borneo or melanesia or papua new guinea or vanuatu or haiti or comoros or madagascar or sri lanka) |
