# Supplementary material for: Financial motivation models for community health workers in low- and middle-income countries: a scoping review
Source: Glob Health Action. 2025 Apr 4;18(1):2480412. doi: 10.1080/16549716.2025.2480412 (PMC11980195; doi:10.1080/16549716.2025.2480412)
Supplement: Supplementary file 3_Strengths and weaknesses_.docx [file ZGHA_A_2480412_SM3205.docx]

# Supplementary file 3 - Strengths and weaknesses of each type of compensation

| ***Strengths*** | |  | ***Weaknesses*** | |  |
| --- | --- | --- | --- | --- | --- |
| Fixed compensation | | | | |  |
|  | Improve performance | [1] | Short-term contracts | [2] |  |
|  |  |  | Lack of incentives affects CHW motivation | [3] |  |
|  | Improve motivation | [4-6] | Low wages | [3, 7] |  |
|  | Add prestige and value of CHWs within the communities | [5] | Irregular payments (especially in the case of NGOs) | [3, 4, 8-12] |  |
|  | Add career development opportunities contributing to CHW retention | [2, 8, 9, 13] | Perception of earning inadequate salaries and/or motivation/incentives for the workload and the environment’s socioeconomic realities | [2, 3, 8, 12-18] |  |
|  | Heighten sense of belonging | [12] | Disparity between CHWs receiving salary-based payments and those relying on a monthly stipend can lead to feelings of frustration | [4] |  |
|  | Improve living conditions through regular and adequate wages | [5, 6, 15, 19] | Salary discrimination between CHW cadres | [18] |  |
|  | Wages actually stand as a leading indicator of the performance, motivation and quality of services rendered by CHWs | [7, 20, 21] | Preference for holding an employee status over a volunteer status due to stability issues | [12, 13] |  |
|  | Cover CHWs work-related expenses | [12] | Confusion as to the limits of the tasks and roles assigned to CHWs | [12] |  |
|  | Include guidance measures such as feedback and non-material incentives | [4, 6, 22, 23] | Confusion as to the possibility of having other jobs at the same time | [12] |  |
|  |  |  | Change in perception regarding their status (community agents versus government employees) | [3] |  |
|  | Appreciation of the salary of CHWs by their family members | [5] | Differential treatment based on the source of compensation | [4] |  |
|  |  |  | Lack of support from family members and neighbors, particularly in the early stages, due to the perceived low honorarium | [6] |  |
| Performance-based compensation | | | | |  |
|  | Improve performance | [24-27] | Imbalance between the number of tasks at hand and the number of paid activities | [11, 28-31] |  |
|  | Improve motivation (income generated by performance bonuses) | [11, 24, 25, 31-34] | Lack of understanding of the compensation system | [33, 35] |  |
|  | Increase interest in work | [31, 36] | Lack of time for family and other income-generating activities | [33] |  |
|  | Create a friendly competition | [27] |  |  |  |
|  | Inspire peer to-peer learning from lower-scoring and higher-scoring CHWs | [27] |  |  |  |
|  | Grant CHWs further autonomy regarding household purchases and management | [24, 25, 32, 37] | Turning down certain non-paid activities to perform paid activities | [3, 33, 37, 38] |  |
|  | CHWs are better able to contribute to health decisions | [37] | Incentives insufficient to cover work-related expenses (e.g., transportation, communication, uniforms) | [37, 39] |  |
|  | CHWs assert greater authority within their household | [32] | Payment worksheet completion challenges | [37] |  |
|  | Strengthen CHWs ties with community members and members and program beneficiaries | [33, 38] | Excessive workload | [32] |  |
|  | Favours low attrition rates | [30] | Create inequalities among CHWs (as remuneration is based on the performance of each individual) | [31, 33] |  |
|  | Less susceptible to fraud, as CHWs are paid based on work completed | [30] | Result-based compensation rather than effort-based compensation | [11, 31, 39] |  |
|  | Career advancement opportunities (skills development, additional certifications, mentoring other CHWs) | [34] | Low wages (insufficient financial compensation) | [29, 30, 34, 35] |  |
|  | Additional measures such as supervision improve quality of service | [34] | Irregular payments | [33, 35, 37, 40, 41] |  |
|  | Recognition and respect from their community and program staff | [34] | Complexity and delays in the payment request process | [31, 33, 35, 37] |  |
|  |  |  | Perception among doctors and nurses that the selection of ASHAs was influenced by favoritism and local leaders | [33] |  |
|  |  |  | Negative perception of low wages by family members | [29, 30, 35] |  |
|  |  |  | Downgrading of work by community members and family due to several indirect charges incurred by CHWs | [33, 35] |  |
|  |  |  | Large income gaps among CHWs | [33, 35] |  |
|  |  |  | Delays in payments | [34] |  |
| Income-generating activities | | | | | |
|  | | Additional measures (supervision, per diems for attendance at training and meetings, supply of materials and equipment) improve motivation, skills and quality of service | [34] | Large income gap among CHWs | [42] |
|  | |  |  | Wide disparity in the success of individual cooperatives in generating sufficient income for CHWs and for broader income-generating activities | [3] |
|  | | User fees allow CHWs to restock their supply inventories with high-quality products | [34] | Imbalanced distribution of time between activities | [12, 42] |
|  | | User fees allow CHWs to buy and make available basic necessities for local families | [34] | Devoting more time to volunteer work and less time to income-generating activities | [12, 42] |
|  | | Recognition and respect from their community and program staff | [34] | Lack of financial support from the State and local communities | [43] |
|  | | Career advancement opportunities (skills development, additional certifications, mentoring other CHWs) | [34] | Inconsistent supervision practices | [34] |
|  | | CHW families' appreciation of wages | [12] | Differences in per diems offered by the various partners demotivates CHWs | [34] |
|  | |  |  | Static role: Lack of career advancement opportunities | [34] |
|  | |  |  | Inconsistent training and reporting forms as source of confusion and frustration among CHWs | [34] |
|  | |  |  | Financial burdens and indebtedness due to contribution to access the loan system was incompatible with CHWs’ standard of living | [43] |
|  | |  |  | IGAs do not benefit from financial support from the State and local authorities | [43] |
| Combined compensation | | | | | |
|  | | Improve performance | [44-46] | Perception among CHWs that the program initiators receive all the credit on the national and international stage | [44] |
|  | | Improve motivation | [44-46] | Perception that the health products sold are expensive | [44] |
|  | | Additional revenue from the sale of health products | [3, 44, 46] | Perception of having low wages as compared to the living standard in the community | [47] |
|  | | Appreciation of monthly motivations | [4, 45] | CHW retention using financial incentives often depends on the living standard in the community and on the possibility for CHWs to perform other activities that generate more income | [47] |
|  | | Higher retention rates | [19, 45] | Sale of health products reduced community support for CHWs | [44] |
|  | | Remuneration encourages quality of service | [44] | Perception of lower income in relation to community standard of living | [47] |
|  | |  |  | Perception of insufficient salary support | [3] |
|  | |  |  |  |  |

## References

[1] Vo LNQ, Forse RJ, Codlin AJ, Vu TN, Le GT, Do GC, et al. A comparative impact evaluation of two human resource models for community-based active tuberculosis case finding in Ho Chi Minh City, Viet Nam. BMC public health. 2020;20(1):1-12.

[2] Chilundo BG, Cliff JL, Mariano AR, Rodriguez DC, George A. Relaunch of the official community health worker programme in Mozambique: is there a sustainable basis for iCCM policy? Health policy and planning. 2015;30 Suppl 2:ii54-ii64.

[3] Perry H. Health for the people: National community health worker programs from Afghanistan to Zimbabwe. Maternal and Child Survival Program; 2020.

[4] Ormel H, Kok M, Kane S, Ahmed R, Chikaphupha K, Rashid SF, et al. Salaried and voluntary community health workers: exploring how incentives and expectation gaps influence motivation. Human resources for health. 2019;17(1):59.

[5] Mbachu C, Etiaba E, Ebenso B, Ogu U, Onwujekwe O, Uzochukwu B, et al. Village health worker motivation for better performance in a maternal and child health programme in Nigeria: a realist evaluation. Journal of Health Services Research & Policy. 2022;27(3):222-31.

[6] Rahman M, Jahir T, Yeasmin F, Begum F, Mobashara M, Hossain K, et al. The Lived Experiences of Community Health Workers Serving in a Large-Scale Water, Sanitation, and Hygiene Intervention Trial in Rural Bangladesh. Int J Environ Res Public Health. 2021;18(7).

[7] Koehn HJ, Zheng S, Houser RF, O'Hara C, Rogers BL. Remuneration systems of community health workers in India and promoted maternal health outcomes: a cross-sectional study. BMC Health Serv Res. 2020;20(1):48.

[8] Chevalier C, Lapo A, O'Brien J, Wierzba TF. Why do village health workers drop out? World health forum. 1993;14(3):258-61.

[9] Zulu JM, Kinsman J, Michelo C, Hurtig AK. Integrating national community-based health worker programmes into health systems: a systematic review identifying lessons learned from low-and middle-income countries. BMC public health. 2014;14:987.

[10] Ejigu Y, Abera N, Haileselassie W, Berhanu N, Haile BT, Nigatu F, et al. Motivation and job satisfaction of community health workers in Ethiopia: a mixed-methods approach. Human resources for health. 2023;21(1):35.

[11] Olaniran A, Madaj B, Bar-Zeev S, Banke-Thomas A, van den Broek N. Factors influencing motivation and job satisfaction of community health workers in Africa and Asia-A multi-country study. Int J Health Plann Manage. 2022;37(1):112-32.

[12] Kelly A, Mitra S, Elung'at J, Songok J, Jackson S, Christoffersen-Deb A. Can the financial burden of being a community health volunteer in western Kenya exacerbate poverty? Health promotion international. 2020;35(1):93-101.

[13] Busza J, Dauya E, Bandason T, Simms V, Chikwari CD, Makamba M, et al. The role of community health workers in improving HIV treatment outcomes in children: lessons learned from the ZENITH trial in Zimbabwe. Health policy and planning. 2018;33(3):328-34.

[14] David F, Chin F. An analysis of the determinants of family planning volunteer workers' performance in Iloilo City. Philippine population journal. 1993;9(1-4):12-25.

[15] Jerome G, Ivers LC. Community health workers in health systems strengthening: a qualitative evaluation from rural Haiti. AIDS (London, England). 2010;24 Suppl 1:S67-72.

[16] Shelley KD, Frumence G, Mpembeni R, Mwinnyaa G, Joachim J, Kisusi HK, et al. "Because Even the Person Living With HIV/AIDS Might Need to Make Babies" - Perspectives on the Drivers of Feasibility and Acceptability of an Integrated Community Health Worker Model in Iringa, Tanzania. International journal of health policy and management. 2019;8(9):538-49.

[17] Arora N, Hanson K, Spicer N, Estifanos AS, Keraga DW, Welearegay AT, et al. Understanding the importance of non-material factors in retaining community health workers in low-income settings: a qualitative case-study in Ethiopia. BMJ Open. 2020;10(10):e037989.

[18] Roy S, Pandya S, Hossain MI, Abuya T, Warren CE, Mitra P, et al. Beyond Institutionalization: Planning for Sustained Investments in Training, Supervision, and Support of Community Health Worker Programs in Bangladesh. Global health, science and practice. 2021;9(4):765-76.

[19] Alam K, Oliveras E. Retention of female volunteer community health workers in Dhaka urban slums: a prospective cohort study. Human resources for health. 2014;12:29.

[20] Alam K, Tasneem S, Huq M. Reservation wage of female volunteer community health workers in Dhaka urban slums: a bidding game approach. Health economics review. 2014;4(1):16.

[21] Kosec K, Avula R, Holtemeyer B, Tyagi P, Hausladen S, Menon P. Predictors of Essential Health and Nutrition Service Delivery in Bihar, India: Results From Household and Frontline Worker Surveys. Global health, science and practice. 2015;3(2):255-73.

[22] Kanté AM, Exavery A, Jackson EF, Kassimu T, Baynes CD, Hingora A, et al. The impact of paid community health worker deployment on child survival: the connect randomized cluster trial in rural Tanzania. BMC Health Serv Res. 2019;19(1):492.

[23] Kok M, Abdella D, Mwangi R, Ntinginya M, Rood E, Gassner J, et al. Getting more than "claps": incentive preferences of voluntary community-based mobilizers in Tanzania. Human resources for health. 2019;17(1):101.

[24] Gadsden T, Jan S, Sujarwoto S, Kusumo BE, Palagyi A. Assessing the feasibility and acceptability of a financial versus behavioural incentive-based intervention for community health workers in rural Indonesia. Pilot Feasibility Stud. 2021;7(1):132.

[25] Sakeah E, Bawah AA, Kuwolamo I, Anyorikeya M, Asuming PO, Aborigo RA. How different incentives influence reported motivation and perceptions of performance in Ghanaian community-based health planning and services zones. BMC Res Notes. 2023;16(1):17.

[26] Mohammed A, Dwomoh D, Nonvignon J. The impact of maternal and child health and nutrition improvement project on maternal health service utilization in Ghana: An Interrupted time series analysis. PLOS Glob Public Health. 2022;2(4):e0000372.

[27] PMI Impact Malaria. Community Health Worker Compensation Schemes Assessment. 2023.

[28] Bettampadi D, Boulton ML, Power LE, Hutton DW. Are community health workers cost-effective for childhood vaccination in India? Vaccine. 2019;37(22):2942-51.

[29] Bhatia K. Community health worker programs in India: a rights-based review. Perspectives in public health. 2014;134(5):276-82.

[30] Miller JS, Musominali S, Baganizi M, Paccione GA. A process evaluation of performance-based incentives for village health workers in Kisoro district, Uganda. Human resources for health. 2014;12:19.

[31] Pani SR, Nallala S, Rout SK, Sundari S, Chokshi M, Mokashi T, et al. Effects of Various Financial and Non-financial Incentives on the Performance of Accredited Social Health Activist: Evidence from Two Selected Districts of Odisha. Journal of Health Management. 2022;24(1):74-86.

[32] Condo J, Mugeni C, Naughton B, Hall K, Tuazon MA, Omwega A, et al. Rwanda's evolving community health worker system: a qualitative assessment of client and provider perspectives. Human resources for health. 2014;12:71.

[33] Saprii L, Richards E, Kokho P, Theobald S. Community health workers in rural India: analysing the opportunities and challenges Accredited Social Health Activists (ASHAs) face in realising their multiple roles. Human resources for health. 2015;13:95.

[34] USAID. Incitatifs offerts aux agents de Santé communautaire à Madagascar : Enseignement retenu. African Strategies for Health 4301 N Fairfax Drive, Arlington, VA 22203, É-U; 2015.

[35] Bhatia K. Performance-based incentives of the ASHA scheme: stakeholders' perspectives. Economic and Political Weekly. 2014:145-51.

[36] Khan MS, Mehboob N, Rahman-Shepherd A, Naureen F, Rashid A, Buzdar N, et al. What can motivate Lady Health Workers in Pakistan to engage more actively in tuberculosis case-finding? BMC public health. 2019;19(1):999.

[37] Sarin E, Lunsford SS, Sooden A, Rai S, Livesley N. The Mixed Nature of Incentives for Community Health Workers: Lessons from a Qualitative Study in Two Districts in India. Frontiers in public health. 2016;4:38.

[38] Scott K, Shanker S. Tying their hands? Institutional obstacles to the success of the ASHA community health worker programme in rural north India. AIDS care. 2010;22 Suppl 2:1606-12.

[39] Hämmerli P, Moukam AD, Wisniak A, Sormani J, Vassilakos P, Kenfack B, et al. "My motivation was to save": a qualitative study exploring factors influencing motivation of community healthcare workers in a cervical cancer screening program in Dschang, Cameroon. Reproductive Health. 2022;19(1):1-13.

[40] Kok M, Lucas S, Otege J, Mkwazu Z, Zuleta I, Smet E, et al. The influence of incentives on community health worker motivation in the provision of family planning. A case of Msalala and Shinyanga Districts, Tanzania. J Public Health Afr. 2021;12(2):1319.

[41] De Mesa RYH, Marfori JRA, Fabian NMC, Camiling-Alfonso R, Javelosa MAU, Bernal-Sundiang N, et al. Experiences from the Philippine grassroots: impact of strengthening primary care systems on health worker satisfaction and intention to stay. BMC Health Services Research. 2023;23(1):117.

[42] Onwujekwe O, Ojukwu J, Shu E, Uzochukwu B. Inequities in valuation of benefits, choice of drugs, and mode of payment for malaria treatment services provided by community health workers in Nigeria. The American journal of tropical medicine and hygiene. 2007;77(1):16-21.

[43] Conté, Samb O, Al. Analyse des effets de l’intervention du projet Bajenu Gox sur l’utilisation des services en SMNI dans les postes de sante d’abattoirs Ndagane, Diaoule, Ndande et Parcelles assainies unite 04. Draft; 2019.

[44] Burkot C, Naidi L, Seehofer L, Miles K. Perceptions of incentives offered in a community-based malaria diagnosis and treatment program in the Highlands of Papua New Guinea. Social science & medicine (1982). 2017;190:149-56.

[45] Kawakatsu Y, Sugishita T, Aiga H, Oruenjo K, Wakhule S, Honda S. Effectiveness of four interventions in improving community health workers' performance in western Kenya: a quasi-experimental difference-in-differences study using a longitudinal data. Primary Health Care Research & Development. 2022;23:1-9.

[46] Tariqujjaman M, Rahman M, Luies SK, Karmakar G, Ahmed T, Sarma H. Unintended consequences of programmatic changes to infant and young child feeding practices in Bangladesh. Maternal & Child Nutrition. 2021;17(2):1-14.

[47] Gazi R, Mercer A, Khatun J, Islam Z. Effectiveness of depot-holders introduced in urban areas: evidence from a pilot in Bangladesh. Journal of health, population, and nutrition. 2005;23(4):377-87.
