# Supplementary material for: Financial motivation models for community health workers in low- and middle-income countries: a scoping review
Source: Glob Health Action. 2025 Apr 4;18(1):2480412. doi: 10.1080/16549716.2025.2480412 (PMC11980195; doi:10.1080/16549716.2025.2480412)
Supplement: Supplementary file 2_Description of studies_.docx [file ZGHA_A_2480412_SM3204.docx]

# Supplementary file: Full description of studies

| **Author/Year** | **Countries** | **Program** | **Design** | **Type of Study** | **Health Context** | **Type of Remuneration** | **Type and Number of Study Participants** |
| --- | --- | --- | --- | --- | --- | --- | --- |
| Alam & Oliveras, 2014 [1] | Bangladesh | BRAC Health Program.Shastya Shebika are BRAC CHWs. | Prospective cohort study design | Evaluative | Maternal, neonatal, and child health. Disseminate healthcare messages, identified pregnancies, brought pregnant women to delivery centres,  accompanied pregnant women during their delivery and provided newborn care. | Combined compensation.  Income-generating activity: sale of medicines and health products (income approximately US$3 per month).  Lending system: They receive small loans to carry out income-generating activities.  Self-reported average monthly income: between US$5.92 and $14)  Monthly average CHW income was US$8.15. | 542 CHWs. Control group: 422 CHWs working with the contracted NGO. Case group: 120 CHWs who dropped out |
| Alam et al., 2014 [2] | Bangladesh | BRAC Health Program.Shastya Shebika are BRAC CHWs. | Case–control study | Interventional | Maternal, neonatal, and child health | Fixed compensation  The average salary for CHWs was US$24.11 | 542 CHWs randomly selected from 1,125 CHWs contracted with the NGO. |
| Arora et al., 2020 [3] | Ethiopia | The national health extension program (HEP) | Qualitative research | Exploratory | Primary healthcare (family planning services, latrine construction and basic preventive and curative services for communicable and non-communicable diseases) | Fixed compensation. Monthly salary | Leavers of health extension worker (HEW) positions (n=20), active HEWs (n=16) and key informants (n=11) in the form of policymakers |
| Bettampadi et al., 2019 [4] | India | National Accredited Social Health Activists (ASHA) CHW Program | Cost-effectiveness analysis (Markov modeling) | Evaluative and prospective | Maternal, neonatal, and child health: measles vaccination | Performance-based compensation. ASHAs receive an incentive of $2.14 (INR 150)* per immunization session. Responsibilities of ASHAs consist of both financially incentivized tasks and non-incentivized tasks. One ASHA is deployed for every 1000 residents. | One selected state: Maharashtra – one village with ASHA and one village without ASHA |
| Bhatia, 2014a  [5] | India | National ASHA CHW Program | Historical review | Descriptive | National CHW Programs (review) | Performance-based compensation. ASHA receive irregular amounts. The ASHA incentive for registration of pregnant women is Rs 10. | Literature review: Relevant government policies, meeting minutes, reports, newspaper articles, and statistics, as well as manual searches, were conducted to find studies on the rights-based aspects of large-scale CHW programs |
| Bhatia, 2014b [6] | India | National ASHA CHW Program | Mixed methods | Descriptive | Maternal, neonatal, and child health. Promoting universal immunization, referral, and escort services for  Reproductive and Child Health (RCH) and other health care programs, and encouraging construction of household toilets | Performance-based compensation | Survey: Six communities, 120 households. Questionnaire: 244 ASHAs. Interviews: 24 ASHAs and 29 ASHA facilitators; 20 interviews at the health service level; five focus groups |
| Burkot et al., 2017 [7] | Papua New Guinea | Marasin Stoa Kipa (MSK) program | Qualitative | Analytical | Community-based malaria management | Fixed compensation (implemented between 2007-2009). Fixed monthly allowance of 40 Kina (approx. US$12.62)  2009: Changed to fee-for-service model. MSKs were initially permitted to charge clients two types of fees: for service and for medication.  2012: Combined compensation - With national policy changes, MSKs charge clients just for service + performance-based payment of 1 Kina (approx. US$0.33 at time of research) for each quality blood smear they produce + *Marasin Stoa (medicine store)*  3T (MS3T) social franchise | 19 semi-structured interviews and seven focus groups, involving 61 CHWs and community members in 13 villages across the Southern Highlands, Hela, and Gulf provinces |
| Busza et al., 2018 [8] | Zimbabwe | Pilot project aimed at enhancing testing and improving treatment of HIV in children (ZENITH) | Longitudinal qualitative study | Descriptive and analytical | HIV management in children | Fixed compensation. Monthly motivation: US$20 | Individual interviews with 19 CHWs delivering the program activities |
| Chevalier et al., 1993 [9] | Papua New Guinea | Village Health Workers Program in the Solomon Islands | Quantitative study | Evaluative | Primary health care | Fixed compensation. The average salary is US$13,667, with variations between provinces. | 130 CHWs, of whom 64 are still active and 66 are no longer working. Non-participants: six in the first group and four in the second. Final numbers: 58 in the first group and 62 in the second. Additionally, two observers (one male and one female) per village were interviewed, totaling 119 villages |
| Chilundo et al., 2015 [10] | Mozambique | Elementary multipurpose agents program | Qualitative retrospective case study | Evaluative | Integrated community case management (iCCM), health promotion, and disease prevention | Fixed compensation. Monthly motivation: US$40 | 21 key informants, including representatives from the Ministry of Health, multilateral and bilateral agencies, and non-governmental organizations in Maputo in 2012 |
| Condo et al., 2014 [11] | Rwanda | Community Health Workers (CHWs) program | Qualitative study | Evaluative, descriptive, and cross-sectional | Community-based nutrition | Performance-based compensation | 108 CHWs and 36 beneficiaries |
| David & Chin, 1993 [12] | Philippines | Programmes Barangay service post officers (BSPOs) and Barangay Health workers (BHWs) | Mixed-method study | Evaluative | Family planning | Fixed compensation. Monthly motivation of P200.00 is shared by two BHWs and P400.00 if it is more than two BHWs, depending on the workplace. | 106 Barangay Service Point Officers and 106 Barangay Health Workers (semi-structured interviews); 29 key informants including City Population Office, City Health Office, Barangay Health Station (BHS) staff, and Barangay leaders (in-depth interviews); 92 married couples of childbearing ages, community midwives, and Barangay Health Workers (focus groups) |
| De Mesa et al., 2023 [13] | Philippines | Philippine Primary Care Studies (PPCS) program | A pretest-posttest design | Evaluative | The Philippine Primary Care Studies (PPCS) program | Performance-based payment. | 207 Health community workers (HCWs) (family physicians, nurses, midwives, community health workers, and staff) from the urban, rural, and remote sites |
| Ejigu et al., 2023 [14] | Ethiopia | The national Health Extension Workers (HEWs) program | Mixed-methods cross-sectional which is nested within a national HEP assessment survey | Evaluative | Primary health care (immunization,  antenatal care, family planning, and postnatal care, among others) | Fixed compensation. Salary and benefit packages | 341 health posts and 584 HEWs |
| Gazi et al., 2005 [15] | Bangladesh | Depot-Holders program | Cross-sectional case-control study | Evaluative | Family planning. Five types of services: reproductive planning, child health, control of communicable diseases, limited curative care, and behaviour change communication | Combined remuneration. They were paid an honorarium of Tk 200-500 per month, a commission of 50% of profits from the sale of commodities and 50% of service charge for customers they referred to the NGO clinics | Approximately 800 were selected from each of the three intervention and comparison areas, with 400 from a group of poor households in Dhaka |
| Gadsden et al., 2021 [16] | Indonesia | SMART*health* programme | Mixed-methods feasibility study | Exploratory and evaluative | Cardiovascular disease (CVD) risk screening and management program: Follow up patients at high risk of CVD. Kaders provide guidance on lifestyle modification, monitor and promote adherence to prescribed medicines and encourage  medicines compliance | Performance-based compensation.  If *Kaders* followed up with 100% of their assigned patients each month, they received 500,000 IDR (USD 34.1). If they did not achieve 100% follow-up, *Kaders* received 16,000 IDR (USD 1.1) per patient followed up. Furthermore, if a Kader attempted to follow up with a patient three times without success, this was counted as a successful follow-up. | 40 *Kaders* participated in the study: 20 in the financial incentive village (Sepanjang) and 20 in the non-financial incentive village (Kepanjen). SMARThealth programme administrators (n = 2) |
| Hämmerli et al., 2022 [17] | Cameroun | Cervical cancer screening program in Dschang | Qualitative phenomenolog-ical research methodology | Exploratory | Health promotion and prevention: Inform women, their partners,  and relatives about cervical cancer and how to prevent it, provide information about the cervical cancer screening program and encourage women to attend for  screening. | Performance-based compensation. CHWs distribute tickets with their name to the women and receive for every woman attending the cervical cancer screening program a remuneration. | 11 community healthcare workers |
| Jerome & Ivers, 2010 [18] | Haiti | Community Health Program in Haiti | Qualitative study | Descriptive | HIV | Fixed compensation. Salary not disaggregated. Salary is estimated at 5.8% of the annual budget of all program activities. | 462 CHWs (19 group interviews conducted; 13 groups of approximately 27 accompanists each, three groups of seven health agents, and three groups of 5 health educators) |
| Kanté et al., 2019 [19] | Tanzania | Community Health Worker Program under the Connect Trial in Tanzania | Quantitative study: Cluster-randomized, unmasked trial. | Evaluative and intervention-oriented | Child health | Fixed compensation. US$ 348.21 annual salary | 50 villages were in the intervention group, and 51 villages were in the comparison group across three districts. The population of children under five years old in the study areas as of July 31, 2011, was 30,524 in the intervention group (51.7%) and 28,569 in the comparison group (48.3%). 142 paid CHWs were deployed in the 50 intervention villages. |
| Kawakatsu et al., 2022 [20] | Kenya | Community Health Strategy  In the strategy, a community unit (CU) is composed of CHWs, community health extension workers (CHEWs), and community health committee (CHC) | Pre-test and post-test design from the longitudinal survey data collected in western Kenya | Evaluative | Primary health care | Combined remuneration. Monthly remunerations and performance-based payments were provided to 81 CHWs by development partners under the agreement with the MoH | 463 CHWs + 5120 mothers |
| Kelly et al., 2020 [21] | Kenya | CHVs in western Kenya - Community health volunteers (CHV) and Community health extension workers (CHEW) | Qualitative study before and after the change in compensation type | Descriptive and evaluative | Primary health care | Fixed compensation before 2013. Monthly motivation approximately UA$20 to $23). Transitioned to a pooled loan system (post-2013).  -GISE (Group Integrated Savings and Empowerment) consisting of 15 to 30 people to create a constitution and learn basic accounting.  -GISE (Group Integrated Savings and Empowerment) groups issue loans to CHWs so that they can start an income-generating activity.  -These loans are at an interest rate of 10%.  -CSOs were required to provide a bi-weekly contribution as a condition of membership | Study was conducted in two sub-counties: Bunyala and Eldoret West.  Two focus groups per study area: In 2013, 35 CHWs participated. In 2015, 25 CHWs participated. Additional individual interviews with four CHEWs, four CHWs, and four NGO managers. |
| Khan et al., 2019 [22] | Pakistan | Pakistan National Lady Health Workers (LHW) Program | Qualitative Study | Evaluative | Tuberculosis. LHW referred a patient to a government health facility who was diagnosed with TB | Performance-based compensation. LHWs are usually responsible for approximately 100–200 households | 12 health program coordinators and 20 Lady Health Workers |
| Koehn et al., 2020 [23] | India | National Programs - ASHA (Accredited Social Health Activists) and Anganwadi Workers (AWWs) | Cross-sectional quantitative study | Evaluative | Maternal and child health | Performance-based compensation – ASHA. ASHAs receive approximately US$10 for facilitating an institutional delivery and US$3 for facilitating a child’s immunization session, though compensation varies by state  Fixed compensation – AWW. Receive a monthly stipend of approximately US$25 and qualify for a government life insurance scheme | 3,455 mothers of children aged six to 23 months |
| Kok et al., 2019 [24] | Tanzania | Community-based mobilizers (CBM) program | Mixed method: Qualitative and quantitative research | Analytical | Sexual and reproductive health services | Fixed compensation. Monthly motivation: 40000 Tsh / US$17.50 per month. | Qualitative interviews: Three focus group discussions with 17 community-based mobilizers (CBMs) and 11 interviews with CBM supervisors and managers. Questionnaires: 61 CBM. |
| Kok et al., 2021 [25] | Tanzania | CHW programmes: NGOs programmes, government through specific (outreach) programmes, World Bank results-based financing (RBF) programme | A qualitative study | Exploratory | Sexual and reproductive health | Performance-based compensation. Financial incentives from  NGOs included per diems and transport allowances for implementing special interventions such as immunizations and other  outreach activities, with a reported maximum monthly compensation of Tsh. 60,000 (US$26). The RBF programme was reported to have a maximum monthly payment of Tsh. 25,000 (US$10.80). | 21 CHWs, 12 supervisors and eight policy makers and NGO representatives |
| Kosec et al., 2015 [26] | India | National Programs: ASHA (Accredited Social Health Activists) and Anganwadi Workers (AWWs) | Quantitative research: Cross-sectional | Analytical | Essential nutrition and health interventions | Performance-based compensation – ASHA. Fixed compensation - AWW | 6,002 households in 400 randomly selected villages in one district of Bihar, as well as an integrated survey of 377 AWW and 382 ASHA from the same villages |
| Mbachu et al., 2022 [27] | Nigeria | The maternal and child health (MCH) component of the Subsidy Reinvestment and Empowerment Programme (SURE-P) | Realist evaluation | Evaluative | Maternal, neonatal, and child health interventions (mobilize pregnant  women, promote antenatal care, encourage facility delivery by a  skilled birth attendant and promote postnatal care for mothers and babies (family planning and immunization), birth preparedness counselling, health education, first aid for minor cuts and injuries, sanitation and hygiene education. | Fixed compensation. Monthly stipend/salary (C/R. A monthly stipend of about US$60 per month | FGDs (n = 32) with eight Village health workers, eight Service users, eight Family members of service users and eight Ward development committees; Interviews (n = 16): six Facility managers (also health workers), 10 other health workers and nine Village health workers |
| Miller et al., 2014 [28] | Uganda | ASC Program Implemented in Kisoro by Partners | Mixed-methods study | Evaluative | Primary healthcare. Follow-up visits and health education activities, health screening and case identifications, improvements in sanitation coverage, and uptake of priority health services (antenatal care, family planning, cervical cancer screening, and childhood immunization) | Performance-based compensation. VHWs are paid based on 20 indicators. They receive from US$0.15 to $1.50, depending on the service. | 34 ASCs who had worked in the program since its inception and eight supervisors |
| Mohammed et al., 2022 [29] | Ghana | The Maternal and Child Health and Nutrition Improvement Project (MCHNP) | Retrospective pre and post-test study design | Evaluative | Maternal, neonatal, and child health interventions, nutrition | Performance-based compensation | 4,713,051 women of reproductive age |
| Olaniran et al., 2022 [30] | Bangladesh, India, Kenya, Malawi and Nigeria | CHW Programs (unspecified if pilot project or national program) | Multiple-case study design using qualitative data | Exploratory | Maternal, neonatal, and child health interventions | Bangladesh: Fixed compensation / India: ASHA = Performance-based compensation; AMN = Fixed compensation / Kenya: Combined compensation / Malawi: Fixed compensation / Nigeria: Fixed compensation | 116 key informant interviews and 32 focus group discussions with 361 individuals across the five countries |
| Onwujekwe et al., 2007 [31] | Nigeria | CHWs program | Multi-method approach | Evaluative | Malaria | Income-generating activities.  Revenues from the sale of antimalarial drugs (chloroquine and sulfadoxine-pyrimethamine). The income from the sale of medicine was about 20,000 naira (US$160) per month. The profit from the profit made was paid to the CSAs in the form of a monthly stipend or commission. Allocations varied per CHW according to the number of patients treated and the income they earned (minimum 200 Naira in one month and maximum more than 700 Naira in one month). The average was more than about 25 naira per month. | Pre-test: 300 household heads. Development of the intervention: A participatory approach based on meetings, feedback sessions, and discussions with community leaders and local and national malaria control officials. Assessments: Discussions with community leaders and half (150) of the household heads interviewed in the pre-test. |
| Ormel et al., 2019 [32] | Bangladesh, Ethiopia, Kenya, Indonesia, Malawi, and Mozambique | CHW programmes: Bangladesh: Family welfare assistants (FWAs); *Shasthya kormis* health volunteers / Ethiopia: HEW health extension workers (HEWs) / Malawi: health surveillance assistant (HSAs) Indonesia: kader volunteer CHWs / Kenya: CHWs / Mozambique: *Agentes polivalentes elementares* (elementary multipurpose agents) (APEs) | Comparative analysis of six qualitative studies | Analytical | Multiple health context: Family planning. Health surveillance, primary health care | Fixed compensation   1. FWA Salary: US$98 per month - Bangladesh 2. SK Salary: US$39 per month - Bangladesh 3. HSA Salary: US$100 per month – Malawi 4. HEW salary: US$46 per month – Ethiopia 5. Indonesia: Allowance: USD5 + material incentive 6. Kenya: CHWs Allowance: USD23 + material incentive 7. Mozambique: APEs Allowance: US$40 + material incentive | 250 interviews with CHWs, healthcare professionals, partner organization teams, and community members |
| Pani et al., 2022 [33] | India | Accredited Social Health Activists (ASHAs) | Cross-sectional qualitative approach | Interpretive structural modelling | Primary healthcare | Performance-based compensation. ASHAs are getting incentives for two segments of activities. The first segment comprises 13 activities for which an ASHA can get a maximum ₹3,500 incentive per month—also called the assured financial incentives. The second segment comprises 40 activities aligned with various health programs and  provides scope to the ASHAs to earn beyond ₹3,500 per month. | 54 informants (ASHAs, auxiliary nurse midwives and block and district levels managers) |
| Rahman et al., 2021 [34] | Bangladesh | WASH-Benefits Bangladesh trial | Qualitative assessment research design | Descriptive | Water, Sanitation, and Hygiene Intervention | Fixed compensation. A monthly honorarium of 1500 BDT per month (equivalent to USD 20) + transportation and training allowances + commodities | 135 CHWs: Nine focus group discussions and 18 in-depth interviews |
| Roy et al., 2021 [35] | Bangladesh | The community health worker (CHW) program | Qualitative approach | Descriptive | Primary healthcare (community health care practitioners) / Immunization and primary health care (health assistants) / Sexual and reproductive health (family welfare assistants) | Fixed compensation. Monthly salary pay scale is dictated by their grade position + allowances for accommodation/housing, transportation, child education, and tiffin (i.e., light meal), as well as for two yearly festivals and the Bengali New Year. | 151 participants: Twenty FGDs were conducted among 121 participants (family welfare assistants, health assistants, and their direct supervisors); 30 interviews were conducted with upazila and district-level stakeholders (i.e., civil surgeon, deputy directors of family planning, upazila health and family planning officers, upazila family planning officer). |
| Sakeah et al., 2023 [36] | Ghana | Korean International Cooperation Agency Community-based Health Planning and Services Strengthening: KOICA CHPS + project | Quasi-experimental study design with post-intervention measurement | Evaluative | Maternal, newborn and child health | Performance-based compensation | 30 In-depth interviews (IDIs) and 31 Focus Group Discussions (FGDs) with health professionals and community members |
| Saprii et al., 2015 [37] | India | ASHA National Program | Qualitative Study | Exploratory | Maternal and Child Health. Facilitating immunization, referral and escort services for institutional deliveries. Each ASHA is meant to cover a population of 1000. | Performance-based compensation. ASHAs are paid Rs. 600 (£6.1 approx.) for every woman who is successfully referred for institutional delivery, and the post-partum mother is also entitled to Rs. 700 (£7.1 approx.) | 36 participants: 18 in-depth interviews and three focus group discussions with 18 participants (ASHAs, key stakeholders and community members). |
| Sarin et al., 2016 [38] | India | ASHA National Program | Qualitative Study | Exploratory | Maternal and Child Health. Counseling pregnant women and facilitating access to antenatal care (ANC) and facility delivery; distributing oral rehydration packets, iron folic acid tablets, chloroquine, oral contraceptive pills, and condoms; facilitating access to immunizations for children; and providing information on health and health practices. | Performance-based compensation. ASHAs receive incentives for the activities they carry out, which range from Rs 50 (US$0.83) for early registration of pregnancy to Rs 1000 (US$16.67) for facilitating permanent contraceptive methods. ASHAs are also paid to identify and refer cases of leprosy, tuberculosis, and malaria, undertake health surveys, and mobilize village health meetings. | 49 eligible ASHAs: For each ASHA, one family member was interviewed, including 34 husbands, 13 mothers-in-law, one sister-in-law, and one son. |
| Scott & Shanker, 2010 [39] | India | ASHA National Program | Qualitative study | Case study | Maternal and Child Health, adolescent health and environmental hygiene. Encourage women to register pregnancies and visit local health centres, escort people to the primary health centre (PHC) as needed, bring children to immunization clinics, encourage family planning, treat basic illness and injury with first aid, keep demographic records and improve village sanitation. | Performance-based compensation. For example, if an ASHA facilitates a birth in a health centre rather than at home, she receives Rs. 600, and the mother receives Rs. 1400. ASHAs also receive Rs. 150 for each child completing an immunization  session and Rs. 150 for each individual who undergoes  surgical sterilization. | 25 participants, including nine ASHAs, and 16 other stakeholders (four local government members, four local individuals, two pharmacists, three doctors, two Anganwadi center staff, and one health worker). |
| Shelley et al., 2019 [40] | Tanzania | TUNAJALI (Swahili for “We Care”) initiative, involving “Wawezeshaji wa Afya ya Jamii (WAJAs)” in Swahili, which translates to “Community Health Agents” | Qualitative Study | Descriptive and exploratory | HIV and Maternal, Neonatal, and Child Health services/Promotion, home-based care | Fixed compensation. Increase in monthly stipend from 35 000 to 40 000 Tanzanian Shillings (~US$17 to US$20) per month | 67 participants: In-depth interviews with 36 CHWs, 21 supervisors, and 10 program managers |
| Tariqujjaman et al., 2021 [41] | Bangladesh | BRAC's MIYCN programme | Mixed method evaluation | Evaluative | Infant and young child feeding (IYCF) practices. Providing essential healthcare services, disseminating messages on maternal  and child nutrition and selling various health products, such as  paracetamol, iron-folic acid, calcium, zinc tablets, oral rehydration salt (ORS), oral contraceptive pills, condoms, pregnancy strips, delivery  kits, sanitary napkins and Pushtikona-5 (a brand of micronutrient powder [MNP]) | Combined compensation (performance-based compensation and income-generating activities).  The incentives included BDT 50.0 (US$0.59) for confirming the breastfeeding of a child within one hour after birth, BDT 20.0 (US$0.24) per month for confirming exclusive breastfeeding of a child for 6 months, BDT 10.0 (US$0.12) for confirming the start of timely complementary feeding, BDT 5.0 (US$0.059) for confirming the start of foods of animal source and BDT 5.0 for confirming hand washing. Incentives for IYCF counselling were removed in 2016  IGA: Four boxes of MNP were provided to CHWs free of charge as a source of resolving funds (resolving funds was among the income generation activities of CHWs) | 2,633 child-caregiver dyads at baseline, 1,762 at midline and 2,084 at endline. |
| Vo et al., 2020 [42] | Vietnam | Community-based active case finding (ACF) project  named PROPER CARE; IMPACT-TB study | Controlled intervention study | Evaluative | Tuberculosis case finding | Fixed compensation. Salary equivalent to US$136/month. Stipend of US$23/month | 321,020 persons were screened in the community. 70,439 were eligible for testing and 1138 of them started TB treatment |
| Zulu et al., 2014 [43] | Zambia | National Community Health Assistant (CHA) Program | Qualitative Study (Phenomenological Approach) | Descriptive | Management of malaria fevers. Promotion of proper food production, basic sanitation, and detecting risk groups for the prevention of common illness. HIV  prevention, treatment and care programs | Fixed compensation. US$220 per month. | 12 Community Health Assistants |

FGDs = Focus Group Discussions; IDIs = In-depth interviews; CHW = Community health worker; ASHA = National Accredited Social Health Activists; NA: Not applicable

# Grey literature

| **Author/Year** | **Countries** | **Program** | **ASC** | **Health Context** | **Type of Remuneration** |
| --- | --- | --- | --- | --- | --- |
| Devlin et al., 2016 [44] | Liberia | CHA program (Community health assistant and Community health volunteer) | CHA: Community health assistant CHV: Community health volunteer | Primary Healthcare | Combined compensation.  CHA: per diems and allowances equivalent to US$70 per month.  CHV: daily allowances and cash payments do not exceed those of the CHA. Including:  - US$5 per day, not exceeding 10 days per month, for health campaigns.  - A fixed rate of up to US$50 per month (proportional to workload) for ongoing specialized or routine activities. |
| Government of Ghana, 2014 [45] | Ghana | National CHW program | National CHW program ASC | Home visits, health promotion, integrated community case management (iCCM), disease surveillance and all Ghana Health Service GHS-approved and sanctioned health intervention activities at the community level as well as treatment of minor ailments | Fixed compensation.  Fixed salary of US$142 per month. |
| Gilmartin et al., 2019 [46] | Burkina Faso | National Community Health Agent Program | National Community Health Agent Program | Curative health services, health promotion, prevention and support services, and referral of patients requiring care to the health and social promotion center. | Fixed compensation. Monthly motivation of 20,000 FCFA per month/ASBC |
| USAID, 2015 [47] | Madagascar | Three CHW programs were conducted in Madagascar: the USAID Mikolo project, the UNICEF Maternal and Neonatal Community Health project, and the Marie Stopes Madagascar project. Programmatic information from another project (Mahefa) was included in the study. | Three CHW programs were conducted in Madagascar: the USAID Mikolo project, the UNICEF Maternal and Neonatal Community Health project, and the Marie Stopes Madagascar project.  Programmatic information from another project (Mahafa) was included in the study. | Primary health care: priority health areas such as maternal and child health; family planning and reproductive health; nutrition; tuberculosis; and water, sanitation, and hygiene services. | Performance-based incentives. Other types of incentives depending on the project.  - USAID Mikolo: Combined compensation = Performance-based incentives + IGAs (credit for some CHW)  - UNICEF Maternal and Newborn Community Health Project: Performance-based incentives  - Marie Stopes Mobile Clinics Madagascar: Performance-based incentives  - USAID Mahefa: Combined compensation = Performance-based incentives + IGAs (for some CHW) |
| Conté & Samb, 2019 [48] | Senegal | Bajenu Gox Program | Bajenu Gox Program | Maternal and child health. Identification and referral of patients, especially in the field of maternal, neonatal and child health (MNCH) | Income-generating activities. Income-generating activities: A project provides the Bajenu Gox with funds to start self-sustaining businesses that they choose to focus on (livestock and food sales, community shop, processing and sale of local products). |
| PMI Impact Malaria, 2023 [49] | Benin  Burkina Faso  Ghana  Mali  Niger  Malawi  Zambia | Benin: Performance-based compensation  Burkina Faso: Fixed compensation and combined compensation (fixed compensation and income-generating activities)  Ghana: fixed compensation  Mali: Fixed compensation  Niger: Fixed compensation  Malawi: Fixed compensation  Zambia: Fixed compensation | National CHW programs | Benin: iCCM package, which includes diarrhea, nutrition and malaria case management  Burkina Faso: prevention, promotion and curative activities across maternal, neonatal,  and child health, nutrition, sexual and reproductive health, mental health, infectious diseases, noncommunicable  diseases and public health safety.  Ghana: primary health care services, including sexual  and reproductive health (i.e. family planning, antenatal car, PMTCT/early infant diagnosis, skilled delivery,  postnatal care, adolescent sexual and reproductive health), child health (i.e. Expanded Programme on  Immunization, Community Integrated Management of Neonatal and Childhood Illnesses); growth  monitoring; disease surveillance and control; treatment of minor ailments, health education and  counselling.  Malawi: prevention and promotion services in addition to select curative services.  Mali: diarrhea, cough  (pneumonia) and nutrition management for children under five (ORS, Zinc, amoxicillin, and Ready to Use  Therapeutic Food provision), family planning (condom, cycle beads, oral contraceptive pill, implant and  injectable provision), malaria case management for people of all ages (RDTs and ACT), HIV/TB case  management (data collection for suspected cases, counselling, and follow-up for treatment adherence for  confirmed cases), WASH surveillance (data collection on hand washing stations, and latrines) and health center  referrals (for mental health, gender-based violence, family planning extensive counselling, danger signs for  children)  Niger: ASC: health care in health huts, such as family  planning, immunization and delivery of uncomplicated births. RComs - health promotion activities and iCCM services (if more than 5 Km from a health facility).  Zambia: Primary Health Care | Benin: Community relay: Performance based  Financing 50, 000 CFA franc per month (approx. US$75), Qualified Health and Community Worker (ASCQ): Performance-based financing 200,000 CFA per month  (approx. US$300) In Benin, a few CBO representatives cite their “PBF system” as a reason for why the community health indicators have increased. Across the country, mayors or leaders in the community announce which CHWs have achieved the highest level of ranking, which creates a friendly competition and inspires peer to-peer learning from lower-scoring and higher-scoring CHWs. It is important to note that this model does not link payment to just one particular service, but instead on the achievement of a range of service delivery targets, while also integrating non-financial, indirect incentives (e.g. monitoring and sharing of data that recognizes achievement).  Burkina Faso: Burkina Faso’s latest national community health strategy (2019) defines ASBC financial compensation as 20,000 CFA franc (approximately US$30) per month. Regardless of where the ASBC is situated *vis à vis* the health center, each ASBC is entitled to the same base amount of financial compensation. However, ASBC who work outside 5km of the health center are also entitled to a percentage of profits from any medicines they sell to the community.51 For activities that are outside of routine work, notably, seasonal health promotion or vaccine campaigns, mass drug administration or trainings implemented by MOH or implementing partners, the ASBC is entitled to a bonus of 3,000 CFA franc (approximately US$5) for each day of the activity.  Ghana: *(Community Health Officers)* Diploma holders: ~1800 GHC (approx. US$160),  Certificate holders: ~1500 GHC (approx. US$130)  Malawi: *Health Surveillance Assistants* Range based on grade:180,000 –287,000 MWK (approx. US$175 -280)  Mali: ≥ 40,000 CFA franc per month (approximately US$70)  Niger: (*Community Health Worker*) 50,000 CFA franc per month (approximately US$80), (*Relay Community Development)* 20,000 CFA franc per month (approximately US$30)  Zambia: *(Community Health Assistants)* 7,548 ZMK (approx. US$380) |
| Perry, 2020 [50] | Bangladesh  Brazil  Ethiopia  Ghana  Guatemala  India  Iran  Kenya  Liberia  Madagascar  Malawi  Mozambique  Myanmar  Nepal  Niger  Nigeria  Pakistan  Rwanda  Sierra Leone  South Africa  Tanzania  Thailand  Zambia  Zimbabwe | Bangladesh: **The BRAC *Shasthya Shebika*** Combined compensation (performance-based compensation and income-generating activities)  SK: Fixed compensation  Brazil: The Kormi Community Health Workers: Fixed compensation  Ethiopia: Fixed compensation  Ghana: Fixed compensation  Guatemala: Fixed compensation  India: ANM, AWW, and VHGs - Fixed compensation for ANM and AWW; ASHA - Performance-based compensation, and in some state, combined compensation (performance-based compensation and fixed compensation)  Iran: combined compensation (performance-based compensation and fixed compensation)  Kenya: fixed compensation, performance-based compensation or combined compensation (performance-based compensation and fixed compensation)  Libera: Fixed compensation and combined compensation (performance-based compensation and fixed compensation) in some counties  Madagascar: Fixed compensation  Malawi: Fixed compensation  Mozambique: Fixed compensation  Myanmar: Fixed compensation (Malaria volunteers) and performance-based compensation (TD volunteers)  Nepal: Income generating activity  Niger: Fixed compensation  Nigeria: Fixed compensation  Pakistan: Combined compensation (fixed compensation and income-generating activities)  Rwanda: Performance-based compensation and in some of the CHW cooperative: income generating activities  Sierra Leone: Fixed compensation  South Africa: Fixed compensation  Tanzania: Fixed compensation  Thailand: Fixed compensation  Zambia: Fixed compensation  Zimbabwe: Fixed compensation | National CHW programs | Bangladesh: sexual and reproductive health, maternal healthcare  Brazil: Primary health care services  Ethiopia: health promotion, disease prevention (including immunization), provision of family planning services, treatment of selected illnesses, and documentation of community health status  Ghana: maternal and reproductive health services, neonatal and child health services, treatment of minor ailments, health education, and referrals, many at the level of the household.  Guatemala: basic health services between team visits by identifying cases for referral, maintaining the community census, epidemiological monitoring, and raising awareness on health issues  Iran: essential health care services, including maternal and child health care, PHC for adults, reproductive health care, and identification and follow-up for important communicable and non-communicable diseases including COVID-19. Behvarzs are also responsible for limited symptomatic treatments along with environmental and occupational health in their area.  Kenya: ICCM  Liberia: a broad range of preventive and curative services  Madagascar: promotion of health of the community and in the prevention and treatment of diseases and conditions.  Malawi: vaccination, growth monitoring, sanitation, water source protection and water treatment, disease surveillance, health and nutrition talks, provision of contraceptives and supervising traditional birth attendants and village health and water committees.  Mozambique: preventive education and health promotion  Myanmar: health promotion, disease surveillance, and referral services along with selected curative services  Nepal: health education and counseling, support for outreach services, distribution of health commodities, and provision of sick-child care  Niger: primary health care services  Nigeria: curative care based on algorithms included in a book of Standing Orders and referral  Pakistan: maternal and child health, health campaigns of all types, community management of TB, and health education about HIV/AIDS.  Rwanda: ASM – maternal health Pairs diagnosis and treatment of childhood illnesses, diagnosis and treatment of malaria for people of all ages, malnutrition screening and referral, provision of contraceptives, and TB treatment.  Sierra Leone: Integrated Community Case Management of Childhood Illness (iCCM); reproductive, maternal, newborn, and child health; and community-based disease surveillance.  South Africa: prevention and promotion, adherence support for chronic lifelong conditions, early identification of ill-health through screening and referral, and basic therapeutic, rehabilitative and palliative care.  Tanzania: health promotion and basic curative services  Thailand: promoting health and preventing diseases as well as in providing basic health services to local communities  Zambia: health promotion and disease prevention. CHAs are also trained in basic curative service, identifying patients who need referral.  Zimbabwe: comprehensive set of services, from health promotion to the provision of services to referral to the next level of care at the local clinic or health center. They also are trained to diagnose and treat common conditions such as diarrhea and malaria. | Bangladesh: they are given small loans to establish revolving funds, which they use to make some money by selling health products at a small mark-up. SSs earn an income from selling supplies such as oral contraceptives, birthing kits, iodized salt, condoms, essential medications, sanitary napkins, and vegetable seeds. They also receive incentives for good performance that is based on achieving specific objectives during that month, such as identifying a certain number of pregnant women during their first trimester.  SKs receive a monthly salary of US$190.  Brazil: CHA receive a salary that ranges from 954 to 1,600 Brazilian *Reais*, or approximately US$226-380 per month. CHAs attribute value to their role when they talk of their close ties to the community, but they complain about their low salary and low status, particularly in comparison with higher-level professionals.  Ethiopia: HEW receive a base salary of approximately US$84 per month  Ghana: CHOs are full-time salaried employees of the Ministry of Health. The starting monthly salary is around 800 Ghana *cedis* (about US$140). Additional incentives for CHOs include extra paid leave days and the opportunity to advance their education with paid educational leave.  Guatemala: government stipend of US$50 per month  India: Auxiliary Nurse-Midwives (ANMs) receive a government salary of approximately US$280 per month.  Accredited Social Health Activists (ASHAs) receive performance-based payments, averaging US$ 42-56 (Rs. 3,000 to 4,000) per month, and in 2018 the Government of India introduced a fixed incentive of approximately US$ 29 per month (Rs. 2,000) for completing routine and recurring tasks, such as convening the monthly village health, sanitation and nutrition committee meeting. Some states have also begun supplementing the income of ASHAs by providing a fixed monthly honorarium from state funds. Also, some states have introduced ASHA motivation and recognition initiatives such as cash awards for the best-performing ASHAs. Challenge: dissatisfaction among ASHAs with their remuneration  Anganwadi Workers (AWWs) receive an “honorarium” of US$50 – 130 per month.  Village Health Guides (VHGs): receive a salary of approximately US$ 24 (in current value) per month. The stipend provided to the VHGs caused them and their communities to think of them not as community agents, but instead as simply another level of government employee. Remuneration also became a large burden for the Central government.  Iran: *Behvarzs and Moraghebe-Salamat* receive a monthly salary of approximately US$ 350 per month from the government. A performance-based formulation of payment is provided as an additional incentive. Commonly cited challenge mentioned by *Behvarzs* include insufficient salary support.  Kenya: receive a salary of approximately US$ 20 – 60 a month. Of 1,090 CHVs interviewed in the Kenya Community Health Assessment,11 34% reported receiving monthly stipends (59% of whom reported that they were paid monthly, and their pay was based on the monthly reports they submitted), while an additional 24% reported that they had signed a performance contract based on certain targets. The remaining stated that they received pay that was based on the achievement of performance targets. The remuneration of CHVs is inadequate and consequently there is also a high drop-out rate of CHVs  Liberia: receive a monthly incentive of US$70 upon submission of their monthly service reports. In some counties, a piloted initiative providing trained traditional midwives with performance-based incentives for antenatal care, postnatal care, and skilled delivery referrals CHSSs receive an incentive of US$ 313 per month  Madagascar: ACNs receive a monthly payment of 50,000 AR (about US$ 17), but only when there is an active project (which is usually financed by an external donor). A recent assessment reported that the financial as well as non-financial incentives for CHWs in Madagascar are found to be unreliable, too dependent on the presence of the project, and inequitably distributed  Malawi: SAs receive a salary of approximately US$63 per month. The performance of CHWs is sub-optimal due to lack of incentives. The lack of incentives affects CHW motivation.  Mozambique: APEs are paid a monthly subsidy (*Meticais* 1,200, which in 2019 was equal to US$ 20) by the Ministry of Health for their services. APEs interviewed in 2013 noted frustration with their subsidy. APEs also anecdotally reported that the Ministry of Health subsidy was not commensurate with the amount of time and effort they spent providing services.  Myanmar: Malaria and TB Volunteers receive monetary incentives with funds from donors. Malaria Volunteers receive the same quarterly incentive and TB Volunteers receive a set amount per TB case referred. Several reviews have suggested problems with performance- or output-based incentives. These contribute to a distortion of health worker priorities, neglect of unpaid tasks, and lack of motivation.28 When the identification or treatment of cases has been monetized and the incidence of disease decreases, motivation declines.  Nepal: An FCHV fund was created by the government of Nepal in 2008–2009; it provided an initial donation of 50,000 Nepali rupees (about US$700) for each of the 3,914 VDCs (further supplemented, in many districts, by development partners). These FCHV funds, managed by the FCHVs themselves, can be accessed for microcredit to support income-generation activities and undertake community development activities. Every year, the government contributes an additional 10,000 rupees (approximately US$140) to these funds.  Niger: ASCs receive a salary of US$100 per month. Volunteers are supposed to receive a monthly incentive of US$20 per month, but this is irregular and infrequent.  Nigeria: CHEW are local government employees and receive approximately US$ 281 per month. Many of the local governments were unable to pay the salaries of the CHEWs  Pakistan: LHWs receive a salary of about US$180 per month. Another incentive they receive is in the form of money that they earn after selling contraceptives to their clients. The LHW stipend is often the only source of family income and is a critical source of family support. Recently, in 2017, LHWs boycotted their participation in a polio campaign because they had not been paid during the previous five months.  Rwanda: two methods of Community Performance-Based Financing (C-PBF): the first is based on CHW cooperative-level achievement of specified targets, and the second (more recently introduced) on individual event-based reporting through a RapidSMS program for cell phones. Anecdotally, CHWs take home around US$ 5-10 on a quarterly basis. There have been some isolated efforts across the country to merge cooperatives in the interest of launching larger-scale business projects. As of now, cooperative income-generation activities (IGAs) range from opening and running small shops to building large-scale farming operations. There is a high degree of variability in terms of (1) the kind of IGAs in which cooperatives are engaged and (2) the degree to which individual cooperatives are successful in generating sufficient income for individual CHWs and for broader income-generating activities.  Sierra Leone: they are "incentivized volunteers and receive an incentive of approximately US$13.50) per month  South Africa: Remuneration levels for these generalist CHWS are in the range of US$150-290 per month. The perceived lower remuneration of the original Home-Based Carers who are not incorporated into the WBPHCPT system is a source of significant local tension,  Tanzania: CHWs were budgeted to receive a salary equivalent to approximately US$140 per month plus benefits. However, the government has not yet begun employing and paying CHWs  Thailand: VHV receives a monthly salary of US$ 20  Zambia: CHAs received a salary of equivalent to US$ 250 per month  Zimbabwe: VHWs receive a quarterly allowance of US$ 42. However, payment is often irregular. Remuneration is inadequate and irregular, which also affects VHWs’ motivation and, hence, their output. |

# References

[1] Alam K, Oliveras E. Retention of female volunteer community health workers in Dhaka urban slums: a prospective cohort study. Human resources for health. 2014;12:29.

[2] Alam K, Tasneem S, Huq M. Reservation wage of female volunteer community health workers in Dhaka urban slums: a bidding game approach. Health economics review. 2014;4(1):16.

[3] Arora N, Hanson K, Spicer N, Estifanos AS, Keraga DW, Welearegay AT, et al. Understanding the importance of non-material factors in retaining community health workers in low-income settings: a qualitative case-study in Ethiopia. BMJ open. 2020;10(10):e037989.

[4] Bettampadi D, Boulton ML, Power LE, Hutton DW. Are community health workers cost-effective for childhood vaccination in India? Vaccine. 2019;37(22):2942-51.

[5] Bhatia K. Community health worker programs in India: a rights-based review. Perspectives in public health. 2014;134(5):276-82.

[6] Bhatia K. Performance-based incentives of the ASHA scheme: stakeholders' perspectives. Economic and Political Weekly. 2014:145-51.

[7] Burkot C, Naidi L, Seehofer L, Miles K. Perceptions of incentives offered in a community-based malaria diagnosis and treatment program in the Highlands of Papua New Guinea. Social science & medicine (1982). 2017;190:149-56.

[8] Busza J, Dauya E, Bandason T, Simms V, Chikwari CD, Makamba M, et al. The role of community health workers in improving HIV treatment outcomes in children: lessons learned from the ZENITH trial in Zimbabwe. Health policy and planning. 2018;33(3):328-34.

[9] Chevalier C, Lapo A, O'Brien J, Wierzba TF. Why do village health workers drop out? World health forum. 1993;14(3):258-61.

[10] Chilundo BG, Cliff JL, Mariano AR, Rodriguez DC, George A. Relaunch of the official community health worker programme in Mozambique: is there a sustainable basis for iCCM policy? Health policy and planning. 2015;30 Suppl 2:ii54-ii64.

[11] Condo J, Mugeni C, Naughton B, Hall K, Tuazon MA, Omwega A, et al. Rwanda's evolving community health worker system: a qualitative assessment of client and provider perspectives. Human resources for health. 2014;12:71.

[12] David F, Chin F. An analysis of the determinants of family planning volunteer workers' performance in Iloilo City. Philippine population journal. 1993;9(1-4):12-25.

[13] De Mesa RYH, Marfori JRA, Fabian NMC, Camiling-Alfonso R, Javelosa MAU, Bernal-Sundiang N, et al. Experiences from the Philippine grassroots: impact of strengthening primary care systems on health worker satisfaction and intention to stay. BMC Health Services Research. 2023;23(1):117.

[14] Ejigu Y, Abera N, Haileselassie W, Berhanu N, Haile BT, Nigatu F, et al. Motivation and job satisfaction of community health workers in Ethiopia: a mixed-methods approach. Human resources for health. 2023;21(1):35.

[15] Gazi R, Mercer A, Khatun J, Islam Z. Effectiveness of depot-holders introduced in urban areas: evidence from a pilot in Bangladesh. Journal of health, population, and nutrition. 2005;23(4):377-87.

[16] Gadsden T, Jan S, Sujarwoto S, Kusumo BE, Palagyi A. Assessing the feasibility and acceptability of a financial versus behavioural incentive-based intervention for community health workers in rural Indonesia. Pilot and feasibility studies. 2021;7(1):132.

[17] Hämmerli P, Moukam AD, Wisniak A, Sormani J, Vassilakos P, Kenfack B, et al. “My motivation was to save”: a qualitative study exploring factors influencing motivation of community healthcare workers in a cervical cancer screening program in Dschang, Cameroon. Reproductive health. 2022;19(1):133.

[18] Jerome G, Ivers LC. Community health workers in health systems strengthening: a qualitative evaluation from rural Haiti. AIDS (London, England). 2010;24 Suppl 1:S67-72.

[19] Kanté AM, Exavery A, Jackson EF, Kassimu T, Baynes CD, Hingora A, et al. The impact of paid community health worker deployment on child survival: the connect randomized cluster trial in rural Tanzania. BMC Health Serv Res. 2019;19(1):492.

[20] Kawakatsu Y, Sugishita T, Aiga H, Oruenjo K, Wakhule S, Honda S. Effectiveness of four interventions in improving community health workers’ performance in western Kenya: a quasi-experimental difference-in-differences study using a longitudinal data. Primary health care research & development. 2022;23:e20.

[21] Kelly A, Mitra S, Elung'at J, Songok J, Jackson S, Christoffersen-Deb A. Can the financial burden of being a community health volunteer in western Kenya exacerbate poverty? Health promotion international. 2020;35(1):93-101.

[22] Khan MS, Mehboob N, Rahman-Shepherd A, Naureen F, Rashid A, Buzdar N, et al. What can motivate Lady Health Workers in Pakistan to engage more actively in tuberculosis case-finding? BMC public health. 2019;19(1):999.

[23] Koehn HJ, Zheng S, Houser RF, O'Hara C, Rogers BL. Remuneration systems of community health workers in India and promoted maternal health outcomes: a cross-sectional study. BMC Health Serv Res. 2020;20(1):48.

[24] Kok M, Abdella D, Mwangi R, Ntinginya M, Rood E, Gassner J, et al. Getting more than "claps": incentive preferences of voluntary community-based mobilizers in Tanzania. Human resources for health. 2019;17(1):101.

[25] Kok M, Lucas S, Otege J, Mkwazu Z, Zuleta I, Smet E, et al. The influence of incentives on community health worker motivation in the provision of family planning. A case of Msalala and Shinyanga Districts, Tanzania. Journal of Public Health in Africa. 2021;12(2).

[26] Kosec K, Avula R, Holtemeyer B, Tyagi P, Hausladen S, Menon P. Predictors of Essential Health and Nutrition Service Delivery in Bihar, India: Results From Household and Frontline Worker Surveys. Global health, science and practice. 2015;3(2):255-73.

[27] Mbachu C, Etiaba E, Ebenso B, Ogu U, Onwujekwe O, Uzochukwu B, et al. Village health worker motivation for better performance in a maternal and child health programme in Nigeria: a realist evaluation. Journal of Health Services Research & Policy. 2022;27(3):222-31.

[28] Miller JS, Musominali S, Baganizi M, Paccione GA. A process evaluation of performance-based incentives for village health workers in Kisoro district, Uganda. Human resources for health. 2014;12:19.

[29] Mohammed A, Dwomoh D, Nonvignon J. The impact of maternal and child health and nutrition improvement project on maternal health service utilization in Ghana: An Interrupted time series analysis. PLOS Global Public Health. 2022;2(4):e0000372.

[30] Olaniran A, Madaj B, Bar‐Zeev S, Banke‐Thomas A, van den Broek N. Factors influencing motivation and job satisfaction of community health workers in Africa and Asia—A multi‐country study. The International Journal of Health Planning and Management. 2022;37(1):112-32.

[31] Onwujekwe O, Ojukwu J, Shu E, Uzochukwu B. Inequities in valuation of benefits, choice of drugs, and mode of payment for malaria treatment services provided by community health workers in Nigeria. The American journal of tropical medicine and hygiene. 2007;77(1):16-21.

[32] Ormel H, Kok M, Kane S, Ahmed R, Chikaphupha K, Rashid SF, et al. Salaried and voluntary community health workers: exploring how incentives and expectation gaps influence motivation. Human resources for health. 2019;17(1):59.

[33] Pani SR, Nallala S, Rout SK, Sundari S, Chokshi M, Mokashi T, et al. Effects of Various Financial and Non-financial Incentives on the Performance of Accredited Social Health Activist: Evidence from Two Selected Districts of Odisha. Journal of Health Management. 2022;24(1):74-86.

[34] Rahman M, Jahir T, Yeasmin F, Begum F, Mobashara M, Hossain K, et al. The lived experiences of community health workers serving in a large-scale water, sanitation, and hygiene intervention trial in rural Bangladesh. International journal of environmental research and public health. 2021;18(7):3389.

[35] Roy S, Pandya S, Hossain MI, Abuya T, Warren CE, Mitra P, et al. beyond institutionalization: planning for sustained investments in training, supervision, and support of community health worker programs in Bangladesh. Global Health: Science and Practice. 2021;9(4):765-76.

[36] Sakeah E, Bawah AA, Kuwolamo I, Anyorikeya M, Asuming PO, Aborigo RA. How different incentives influence reported motivation and perceptions of performance in Ghanaian community-based health planning and services zones. BMC Research Notes. 2023;16(1):17.

[37] Saprii L, Richards E, Kokho P, Theobald S. Community health workers in rural India: analysing the opportunities and challenges Accredited Social Health Activists (ASHAs) face in realising their multiple roles. Human resources for health. 2015;13:95.

[38] Sarin E, Lunsford SS, Sooden A, Rai S, Livesley N. The Mixed Nature of Incentives for Community Health Workers: Lessons from a Qualitative Study in Two Districts in India. Frontiers in public health. 2016;4:38.

[39] Scott K, Shanker S. Tying their hands? Institutional obstacles to the success of the ASHA community health worker programme in rural north India. AIDS care. 2010;22 Suppl 2:1606-12.

[40] Shelley KD, Frumence G, Mpembeni R, Mwinnyaa G, Joachim J, Kisusi HK, et al. "Because Even the Person Living With HIV/AIDS Might Need to Make Babies" - Perspectives on the Drivers of Feasibility and Acceptability of an Integrated Community Health Worker Model in Iringa, Tanzania. International journal of health policy and management. 2019;8(9):538-49.

[41] Tariqujjaman M, Rahman M, Luies SK, Karmakar G, Ahmed T, Sarma H. Unintended consequences of programmatic changes to infant and young child feeding practices in Bangladesh. Maternal & Child Nutrition. 2021;17(2):e13077.

[42] Vo LNQ, Forse RJ, Codlin AJ, Vu TN, Le GT, Do GC, et al. A comparative impact evaluation of two human resource models for community-based active tuberculosis case finding in Ho Chi Minh City, Viet Nam. BMC public health. 2020;20:1-12.

[43] Zulu JM, Kinsman J, Michelo C, Hurtig AK. Integrating national community-based health worker programmes into health systems: a systematic review identifying lessons learned from low-and middle-income countries. BMC public health. 2014;14:987.

[44] Devlin K, Egan KF, Pandit-Rajani T. Community Health Systems Catalog Country Profile: Liberia. Retrieved from Arlington, VA. 2016.

[45] Government of Ghana. National Community Health Worker (CHW) Program. 2014.

[46] Ministère de la santé du Burkina Faso. Dossier d'Investissement en Sante Communautaire au Burkina Faso 2019 - 2023. 2019.

[47] USAID. Incitatifs offerts aux agents de Santé communautaire à Madagascar : Enseignement retenu. African Strategies for Health 4301 N Fairfax Drive, Arlington, VA 22203, É-U; 2015.

[48] Conté, Samb O, Al. Analyse des effets de l’intervention du projet Bajenu Gox sur l’utilisation des services en SMNI dans les postes de sante d’abattoirs Ndagane, Diaoule, Ndande et Parcelles assainies unite 04. Draft; 2019.

[49] PMI Impact Malaria. Community Health Worker Compensation Schemes Assessment. Final Report. <https://assets.speakcdn.com/assets/2594/english_chw_compensation_assessment_final_june_2023_reviewed-2023070602541803.pdf>. 2023.

[50] Perry H. Health for the people: National community health worker programs from Afghanistan to Zimbabwe. Maternal and Child Survival Program; 2020.
